# Supplementary material for: Circuits and mechanisms for TMS-induced corticospinal waves: Connecting sensitivity analysis to the network graph
Source: PLoS Comput Biol. 2024 Dec 5;20(12):e1012640. doi: 10.1371/journal.pcbi.1012640 (PMC11651605; doi:10.1371/journal.pcbi.1012640)
Supplement: S1 Appendix — Fig A. Activation and synaptic weight scalar parameters for the unified model. Fig B. Conduction velocities and afferent activation delays for the unified model. Fig C. Synaptic noise firing rates and synaptic noise weights for the unified model. Fig D. Characterizations of convergence from optimization. Fig E. Visualization of reduced pareto front. Fig F. Effect sizes for all parameters and all waves. Fig G. Conduction delay histograms resulting from orthodromic and antidromic propagation of action potentials. Table A. List of Optimized Synaptic Weight Parameters. Table B. List of Optimized Delay, Activation, and Noise Parameters. (PDF) [file pcbi.1012640.s001.pdf]

S1 APPENDIX

Fig A

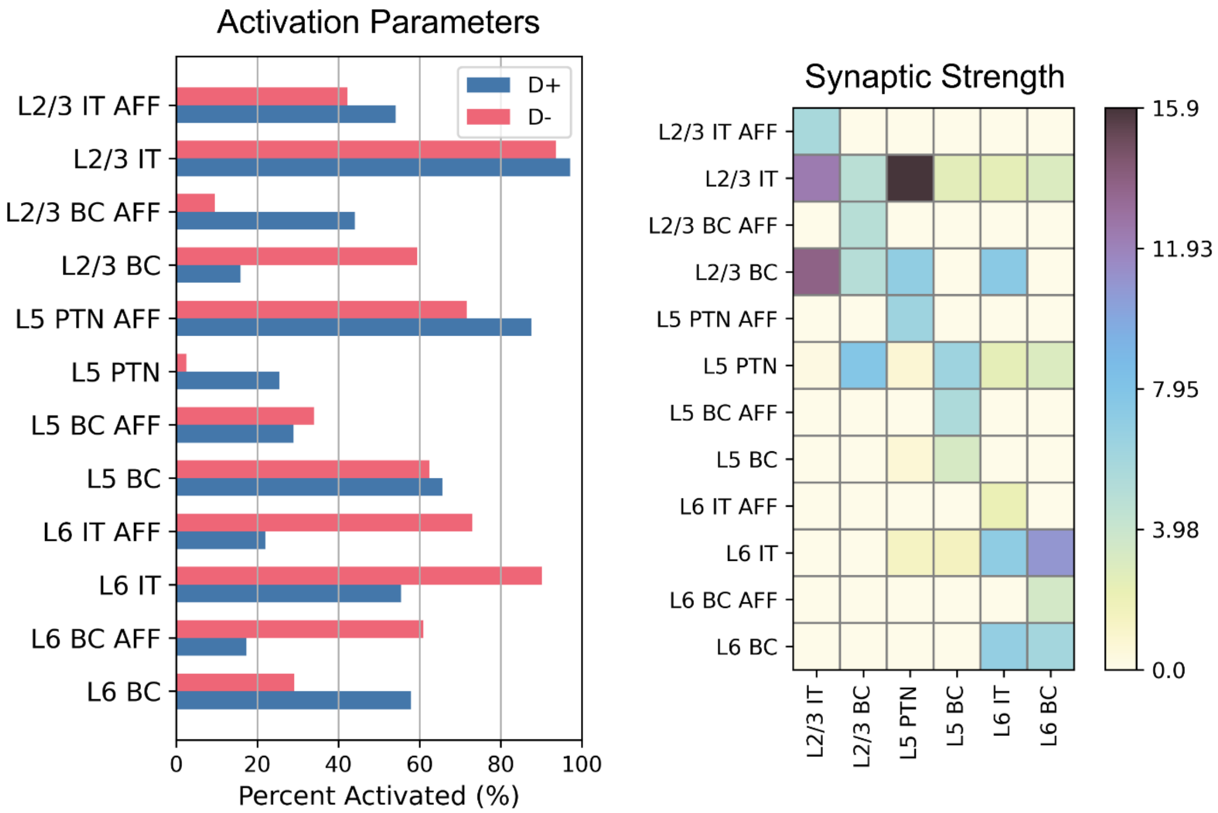

Fig A. Activation and synaptic weight scalar parameters for the unified model.

Activation parameters control the proportion of a cell population that generates an action potential in response to the TMS stimulus. Synaptic strengths of the unified model are on the right. Presynaptic neurons are arranged on the y-axis, and their maximal synaptic conductance on their postsynaptic targets along the x-axis are coded according to the color bar. IT: Intratelencephalic neuron. PTN: Pyramidal tract neuron. BC: Basket cell. AFF: Afferent.

Fig B

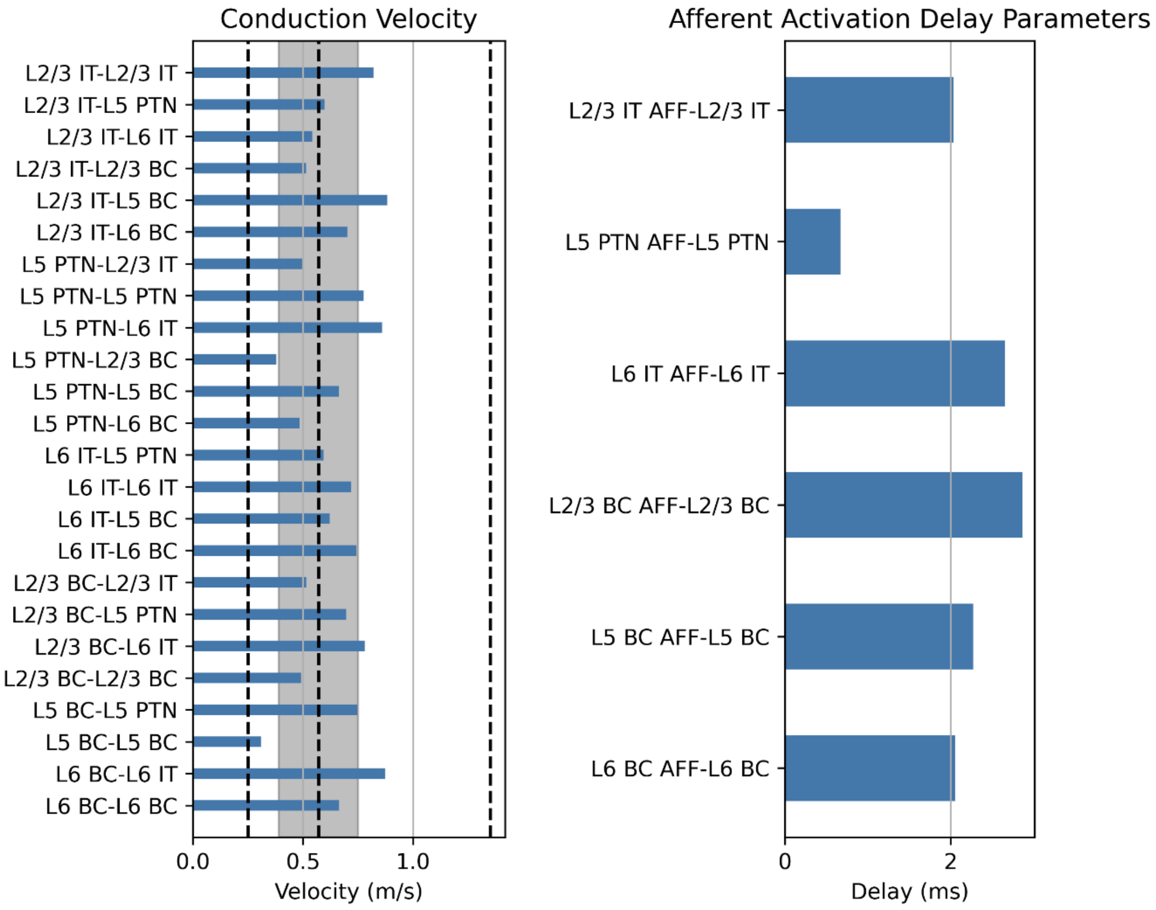

Fig B. Conduction velocities and afferent activation delays for the unified model.

IT: Intratelencephalic neuron. PTN: Pyramidal tract neuron. BC: Basket cell. AFF: Afferent.

Fig C

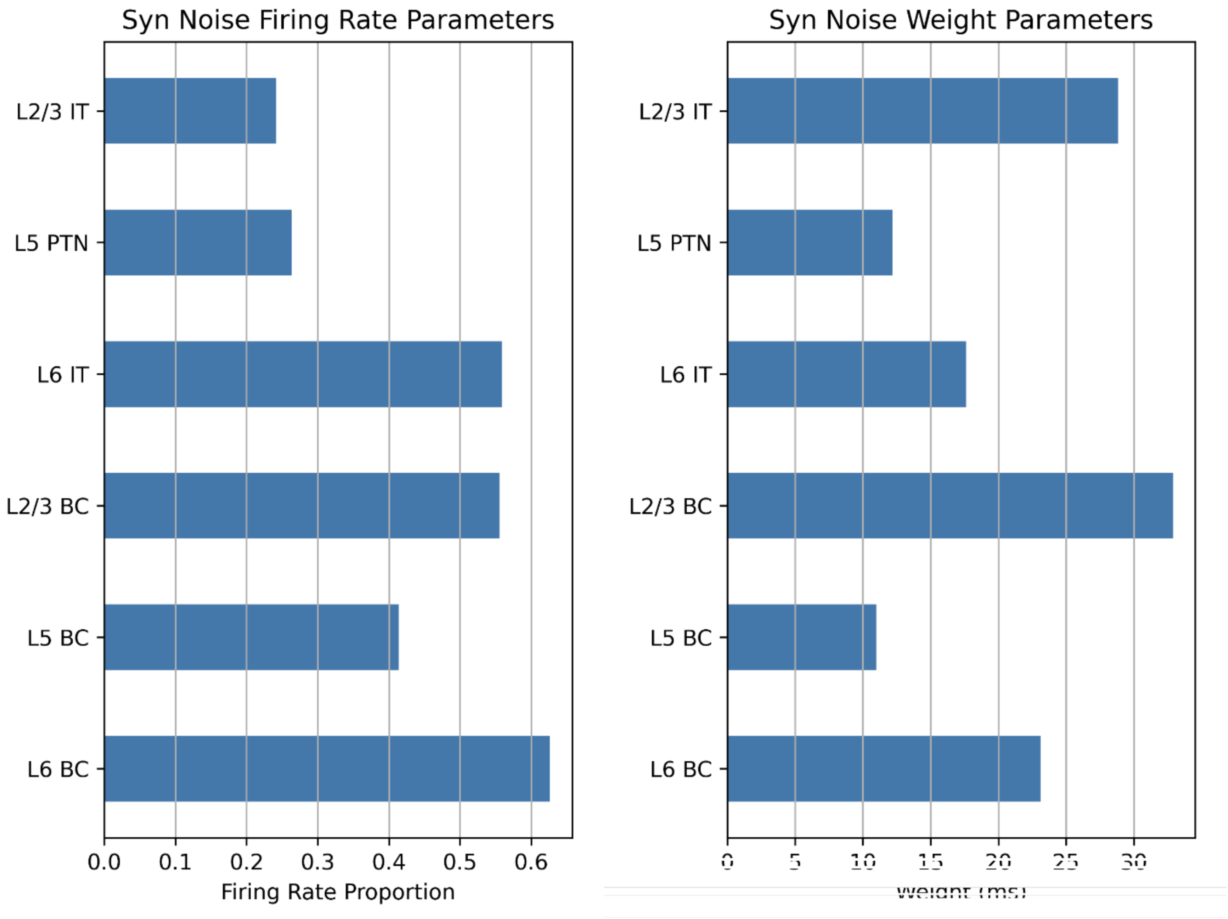

Fig C. Synaptic noise firing rates and synaptic noise weights for the unified model.

IT: Intratelencephalic neuron. PTN: Pyramidal tract neuron. BC: Basket cell. AFF: Afferent.

Fig D

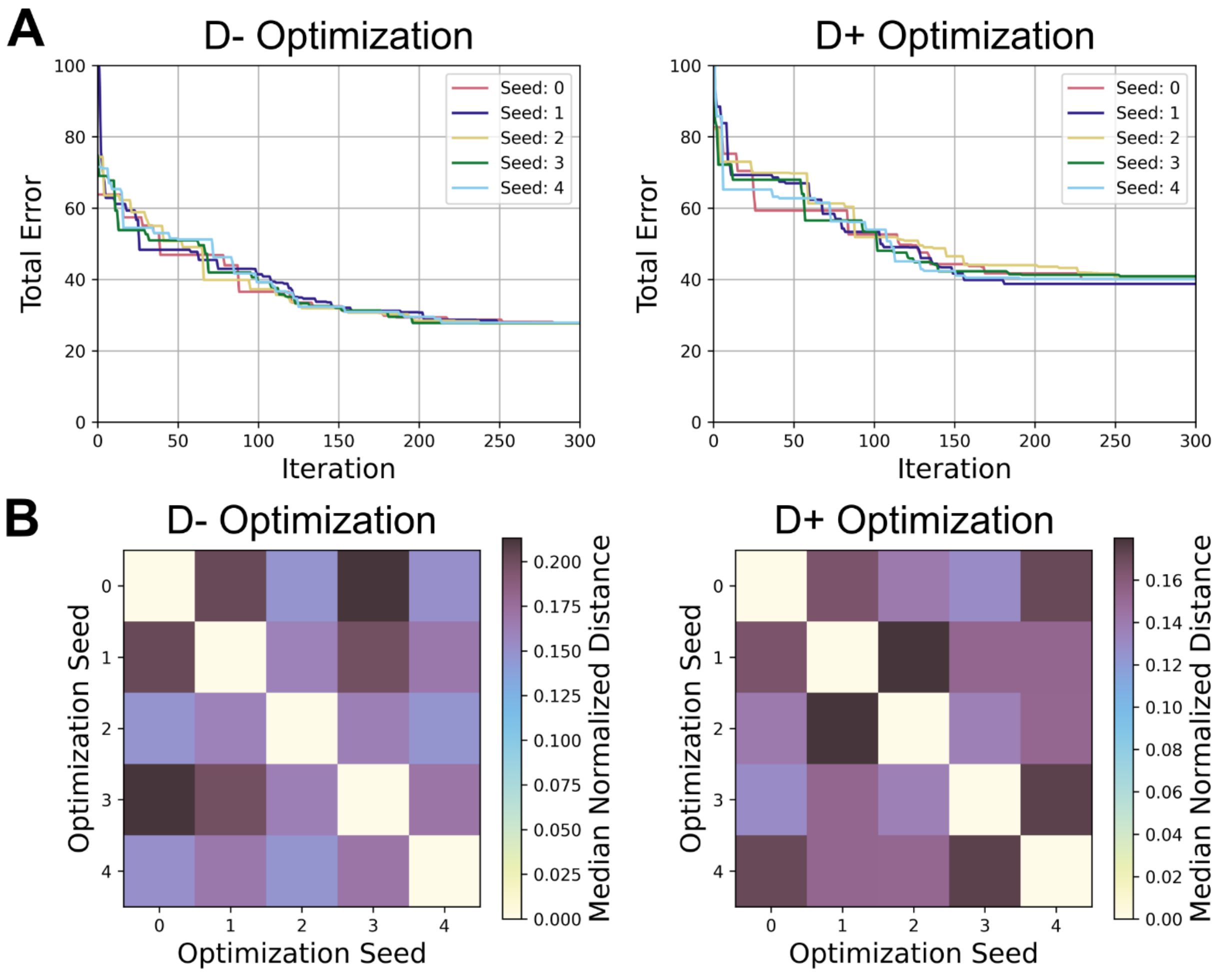

Fig D. Characterizations of convergence from optimization.

A) The cumulative lowest total error is plotted as a function of optimization iteration. Four evolution seeds were run for each responder type (D+ and D−). All seeds converged to errors of similar magnitude. B) The normalized distances of the parameters of the best solutions for each optimization run. The Euclidean distance of the best solutions was normalized by the maximum possible distance given the bounds of the explored parameter space. The diagonals of the matrix are zero because they represent the distance between a solution and itself. The overall normalized distances were 15.5 to 17.2% for D+ and D-, respectively.

Fig E

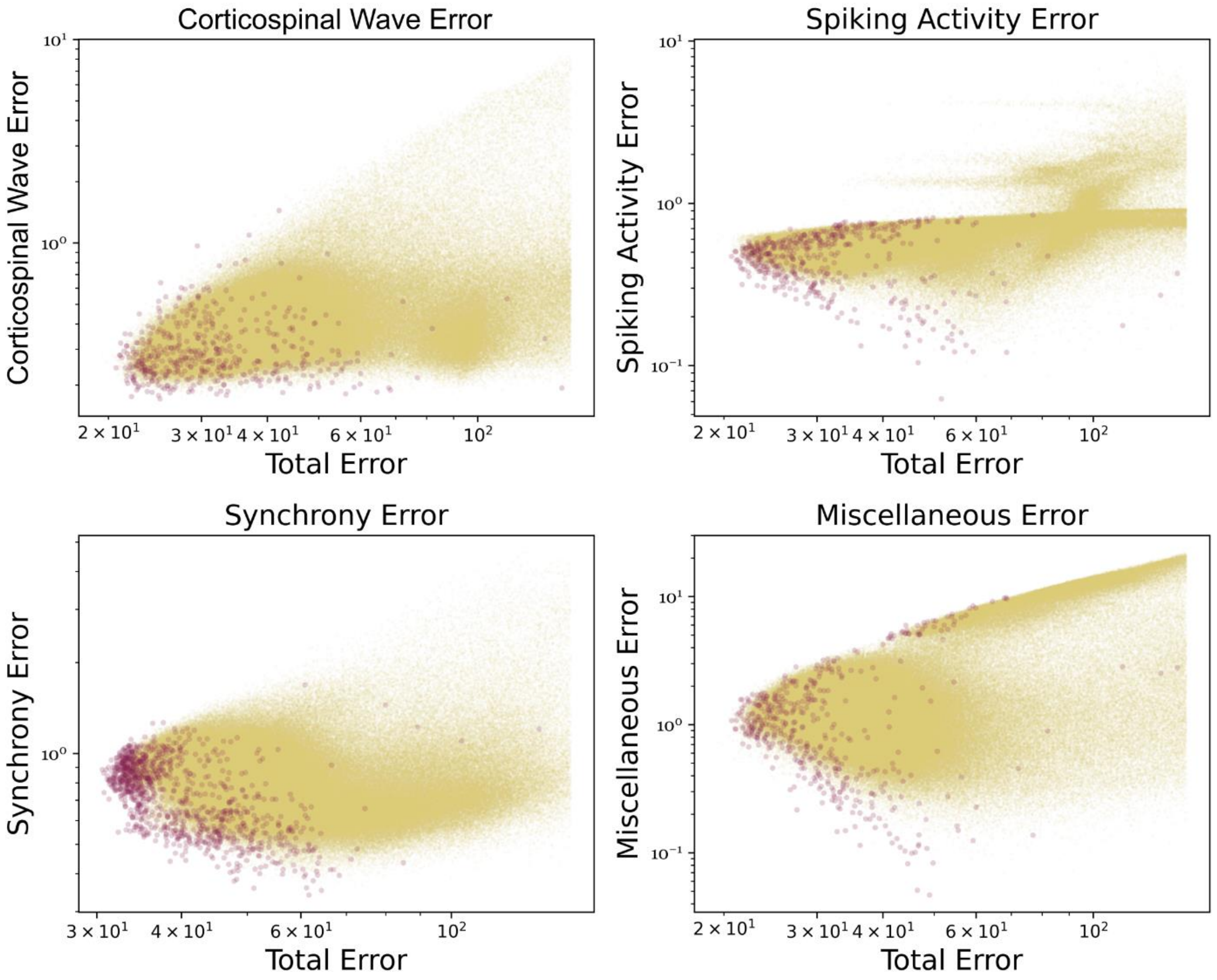

Fig E. Visualization of reduced pareto front.

Objectives were grouped into categories, and their combined error was plotted against the total error. Red dots indicate particles that were pareto dominant, and yellow particles indicate the remaining particles. The correlation between the category error and the total error is a representation of the pareto front and how the category error changes as total error is minimized.

Fig F

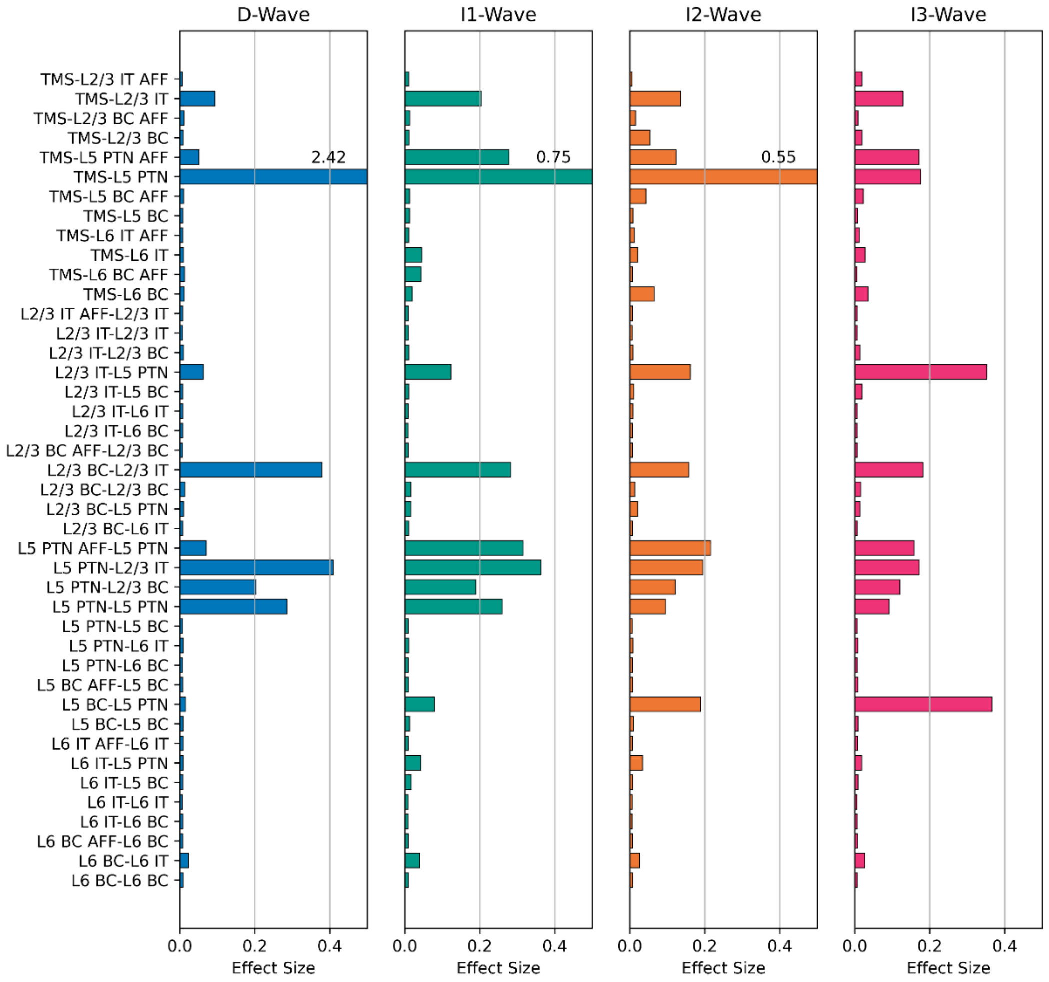

Fig F. Effect sizes for all parameters and all waves.

Effect size was calculated as the integrals of the absolute values of the partial derivatives of the polynomial fits to the TVAT surfaces. Effect sizes were not normalized, and the x-axis maximum was chosen to allow visualization of the smaller effect sizes. Effect sizes that are greater than the x-axis maximum have their values listed above their corresponding bars. Y-axis labels are shared across subplots. IT: Intratelencephalic neuron. PTN: Pyramidal tract neuron. BC: Basket cell. AFF: Afferent.

Fig G

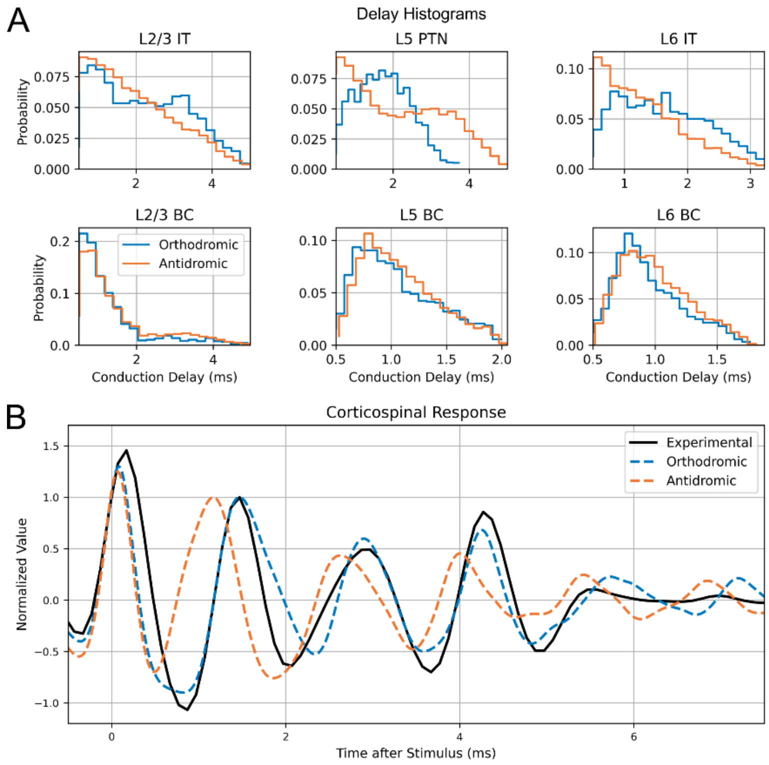

Fig G. Conduction delay histograms resulting from orthodromic and antidromic propagation of action potentials.

Orthodromic propagation used the cell body as the initiation point to compute conduction delays. Antidromic propagation randomly selected a single presynaptic terminal to be activated. The activation then propagated antidromically to activate all other terminals connected to the same axon. A) The resulting conduction delay histograms across all neurons of each cell type are plotted. B) The corticospinal response of the optimized model with each activation type is shown. Orthodromic and antidromic activation result in similar histograms except for L5 PTN and L6 IT with the orthodromic-based delays exhibiting a later peak compared to antidromic-based delay. The effect on the corticospinal response in the model is that the I-waves are similarly proportionally delayed and is consistent with the longer latencies in the L5 PTN conduction delay histogram.

Table A

Table A. List of Optimized Synaptic Weight Parameters

Synaptic Weight Parameters	
L2/3 IT to L2/3 IT (AMPAR/NMDAR)	L2/3 BC to L5 PTN (GABA$_B$R)
L2/3 IT to L5 PTN (AMPAR/NMDAR)	L2/3 BC to L6 IT (GABA$_A$R)
L2/3 IT to L6 IT (AMPAR/NMDAR)	L2/3 BC to L6 IT (GABA$_B$R)
L2/3 IT to L2/3 BC (AMPAR/NMDAR)	L2/3 BC to L2/3 BC (GABA$_A$R)
L2/3 IT to L5 BC (AMPAR/NMDAR)	L2/3 BC to L2/3 BC (GABA$_B$R)
L2/3 IT to L6 BC (AMPAR/NMDAR)	L5 BC to L5 PTN (GABA$_A$R)
L5 PTN to L2/3 IT (AMPAR/NMDAR)	L5 BC to L5 PTN (GABA$_B$R)
L5 PTN to L5 PTN (AMPAR/NMDAR)	L5 BC to L5 BC (GABA$_A$R)
L5 PTN to L6 IT (AMPAR/NMDAR)	L5 BC to L5 BC (GABA$_B$R)
L5 PTN to L2/3 BC (AMPAR/NMDAR)	L6 BC to L6 IT (GABA$_A$R)
L5 PTN to L5 BC (AMPAR/NMDAR)	L6 BC to L6 IT (GABA$_B$R)
L5 PTN to L6 BC (AMPAR/NMDAR)	L6 BC to L6 BC (GABA$_A$R)
L6 IT to L5 PTN (AMPAR/NMDAR)	L6 BC to L6 BC (GABA$_B$R)
L6 IT to L6 IT (AMPAR/NMDAR)	L2/3 IT AFF to L2/3 IT (AMPAR/NMDAR)
L6 IT to L5 BC (AMPAR/NMDAR)	L2/3 BC AFF to L2/3 BC (AMPAR/NMDAR)
L6 IT to L6 BC (AMPAR/NMDAR)	L5 PTN AFF to L5 PTN (AMPAR/NMDAR)
L2/3 BC to L2/3 IT (GABA$_A$R)	L5 BC AFF to L5 BC (AMPAR/NMDAR)
L2/3 BC to L2/3 IT (GABA$_B$R)	L6 IT AFF to L6 IT (AMPAR/NMDAR)
L2/3 BC to L5 PTN (GABA$_A$R)	L6 BC AFF to L6 BC (AMPAR/NMDAR)

IT: Intratelencephalic neuron. PTN: Pyramidal tract neuron. BC: Basket cell. AFF: Afferent.

Table B

Table B. List of Optimized Delay, Activation, and Noise Parameters

Parameters	
Conduction velocity scalar L2/3 IT to L2/3 IT	Activation propagation delay stdev. L2/3 IT AFF
Conduction velocity scalar L2/3 IT to L5 PTN	Activation propagation delay stdev. L2/3 BC AFF
Conduction velocity scalar L2/3 IT to L6 IT	Activation propagation delay stdev. L5 PTN AFF
Conduction velocity scalar L2/3 IT to L2/3 BC	Activation propagation delay stdev. L5 BC AFF
Conduction velocity scalar L2/3 IT to L5 BC	Activation propagation delay stdev. L6 IT AFF
Conduction velocity scalar L2/3 IT to L6 BC	Activation propagation delay stdev. L6 BC AFF
Conduction velocity scalar L5 PTN to L2/3 IT	Proportion activated L2/3 IT
Conduction velocity scalar L5 PTN to L5 PTN	Proportion activated L2/3 BC
Conduction velocity scalar L5 PTN to L6 IT	Proportion activated L5 PTN
Conduction velocity scalar L5 PTN to L2/3 BC	Proportion activated L5 BC
Conduction velocity scalar L5 PTN to L5 BC	Proportion activated L6 IT
Conduction velocity scalar L5 PTN to L6 BC	Proportion activated L6 BC
Conduction velocity scalar L6 IT to L5 PTN	Proportion activated L2/3 IT AFF
Conduction velocity scalar L6 IT to L6 IT	Proportion activated L2/3 BC AFF
Conduction velocity scalar L6 IT to L5 BC	Proportion activated L5 PTN AFF
Conduction velocity scalar L6 IT to L6 BC	Proportion activated L5 BC AFF
Conduction velocity scalar L2/3 BC to L2/3 IT	Proportion activated L6 IT AFF
Conduction velocity scalar L2/3 BC to L5 PTN	Proportion activated L6 BC AFF
Conduction velocity scalar L2/3 BC to L6 IT	Noise weight L2/3 IT
Conduction velocity scalar L2/3 BC to L2/3 BC	Noise weight L2/3 BC
Conduction velocity scalar L5 BC to L5 PTN	Noise weight L5 PTN
Conduction velocity scalar L5 BC to L5 BC	Noise weight L5 BC
Conduction velocity scalar L6 BC to L6 IT	Noise weight L6 IT

Conduction velocity scalar L6 BC to L6 BC	Noise weight L6 BC
Activation propagation delay L2/3 IT AFF	Noise rate L2/3 IT
Activation propagation delay mean L2/3 BC AFF	Noise rate L2/3 BC
Activation propagation delay mean L5 PTN AFF	Noise rate L5 PTN
Activation propagation delay mean L5 BC AFF	Noise rate L5 BC
Activation propagation delay mean L6 IT AFF	Noise rate L6 IT
Activation propagation delay mean L6 BC AFF	Noise rate L6 BC

IT: Intratelencephalic neuron. PTN: Pyramidal tract neuron. BC: Basket cell. AFF: Afferent.
