# Supplementary material for: Circuits and mechanisms for TMS-induced corticospinal waves: Connecting sensitivity analysis to the network graph
Source: PLoS Comput Biol. 2024 Dec 5;20(12):e1012640. doi: 10.1371/journal.pcbi.1012640 (PMC11651605; doi:10.1371/journal.pcbi.1012640)
Supplement: Attachment — Submitted filename: response_to_reviewers_iwavemodeling.pdf [file pcbi.1012640.s002.pdf]

PCOMPBIOL-D-24-00373
"Circuits and mechanisms for TMS-induced corticospinal waves: Connecting sensitivity analysis to the network graph"

Response to Reviewers

Several Reviewers' comments were addressed by modifying the optimization methods and regenerating the results. These updates did not significantly affect the major conclusions of the paper related to the structural analysis. However, the new optimization method is more robust.

1. To address one of the comments of Reviewer 2, the model optimization and analysis was re-run with a shorter time-step of 0.01 ms reduced from the original 0.025 ms.

2. In addition, a regularization step was added to the end of the model optimization. Given that some parameters did not greatly affect I-waves, the regularization step reduced the magnitude of low-influence parameters to avoid high-value but low-influence model parameters.

3. The modifications in simulation and optimization resulted in models that have some differences in parameters compared to the original results. However, similarities persist in the parameters and the sensitivity analysis. Furthermore, the conclusions from the structural analysis remain unchanged. This demonstrates that the proposed analysis pipeline is robust, across both time-steps and models.

4. References to the revised manuscript figures are denoted by Fig. 1, 2, etc. Figures specifically made for this response document are referred to as RFig. 1, 2. etc.

Reviewer #1

I am not an expert in the computational methods used in this paper and therefore my comments are directed at the neurophysiological details and conclusions. Physiologically the contribution of the paper is moderate since, as the authors acknowledge, some of the outcomes (e.g. the role of GABAa inhibitory neurons in the I1-wave) do not match known physiology. Its main impact, I think, will be on how this type of relatively unconstrained model with so many adjustable parameters can nevertheless generate some very reasonable conclusions. It is also the first paper to model explicitly the actual outcome of experiments conducted in humans. In this respect it is very impressive. On the negative side, the results are limited to the response of the model to a single stimulus of a single intensity. Behaviour of I-waves at different intensities and in response to double pulse experiments is already known in the physiological literature, so in theory these could be modelled in future papers (see limitations section of the present

paper).
I have the following comments for the authors.

1) I was slightly surprised to see that despite the fact that recordings were taken from sites several cm apart (C1-C2 versus C3-C5), that the latencies of the I-waves in Fig 1B were so similar. Surely the C3-C5 site should be slightly delayed with respect to the C1-C2 site?

We agree with the Reviewer that differences in conduction distance should be reflected in latencies, and the measurements recorded at C3-C5 were delayed by an average of 0.2 ms with respect to the measurements at C1-C2. Given the vertebral distance in for humans at 15 mm [1] and the median conduction velocity of 60 m/s recorded from macaque monkey [2], the estimated delay between C1 and C3 would be 0.5 ms. However, there is a large variability in the conduction velocities (55-75 m/s). Taking into account the entire CST path length from motor cortex to the C1 and C3 (167 and 197 mm, respectively) [1,3], there is a significant overlap in the possible corticospinal latencies (2.2-3 ms at C1 and 2.6-3.6 ms at C3). These sources of variability, including path length variability, likely account for the small latency differences in the present data.

To clarify this for all readers, the following text was added to Experimental Data:

Lines 655-660
"The differences in recording locations result in an average delay of 0.2 ms for the D- subject who was recorded at C3-C5 compared to the D+ subject who was recorded at C1-C2. These delays were appropriate based on estimates of corticospinal conduction velocity and path distances from motor cortex to C1-C5."

References
[1] Cadotte DW, Cadotte A, Cohen-Adad J, Fleet D, Livne M, Wilson JR, et al. Characterizing the Location of Spinal and Vertebral Levels in the Human Cervical Spinal Cord. American Journal of Neuroradiology. 2015 Apr 1;36(4):803–10.

[2] Edgley SA, Eyre JA, Lemon RN, Miller S. Comparison of activation of corticospinal neurons and spinal motor neurons by magnetic and electrical transcranial stimulation in the lumbosacral cord of the anaesthetized monkey. Brain. 1997 May 1;120(5):839–53.

[3] Dalamagkas K, Tsintou M, Rathi Y, O'Donnell LJ, Pasternak O, Gong X, et al. Individual variations of the human corticospinal tract and its hand-related motor fibers using diffusion MRI tractography. Brain Imaging and Behavior. 2020 Jun 1;14(3):696–714.

2) I have presumed that the TMS pulse activates neurons at presynaptic boutons and not cell bodies (hence excludes axon conduction times), apart from TMS activation of PTN neurons which I suppose activates the axon hillock. Is this correct?

We agree with the Reviewer, based on the results of simulation studies, that TMS activation of neurons occurs primarily in axon terminals, and we highlight details of two representative studies that motivated our implementation of activation. In short, these studies demonstrate that the main site of activation is likely the primary axon, but uncertainties remain whether the axon terminals branching from the axon collateral or the portion of the axon that enters and turns within the white matter are more important. To assess the functional consequences of activation in along the primary axon or at presynaptic boutons, we computed the histograms of the conduction delays that were produced using an orthodromic vs antidromic model. We found that for most cell types, the differences in conduction delays were negligible, and differences that did arise would be straightforward changes in the timings of the I-waves, that would be compensated for by the optimization process to reproduce the experimental data.

Salvador et al., 2011 [4] had pyramidal tract neuron models with main axons that extended into the white matter and simplified local axon collaterals. Aberra et al., 2020 [5] had anatomically realistic local axon collaterals and a main axon that extended primarily toward the white matter, but the main axon was truncated and did not enter the white matter. In [4], studies showed that L5 PTNs had lower thresholds in the main axon where it entered the grey matter and turned towards the internal capsule. In [5], the axon terminals had the lowest thresholds but the orientation threshold map had a significant component aligned with the main axon. When the main axon was removed, the orientation threshold map was significantly affected along the orientation aligned with where the main axon used to be. This effect was also consistent for L6 ITs. The importance of the main axon motivated the computation of L5 PTN conduction delays using the soma as a reference point, with uncertainty in its exact initiation point along its path to the white matter to be solved as an open parameter during optimization, i.e., the conduction velocity.

However, the prior interpretation does not apply to the remaining cell types. To characterize the functional difference between orthodromic vs antidromic activation, we computed the population conduction delay histograms for each approach. We labelled the original method as orthodromic which used the cell body as the initiation point. We labelled the second method as antidromic, and in this approach randomly selected a single presynaptic terminal to be activated. The activation then propagated antidromically to activate all other terminals connected to the same axon. The histograms are shown in RFig. 1A.

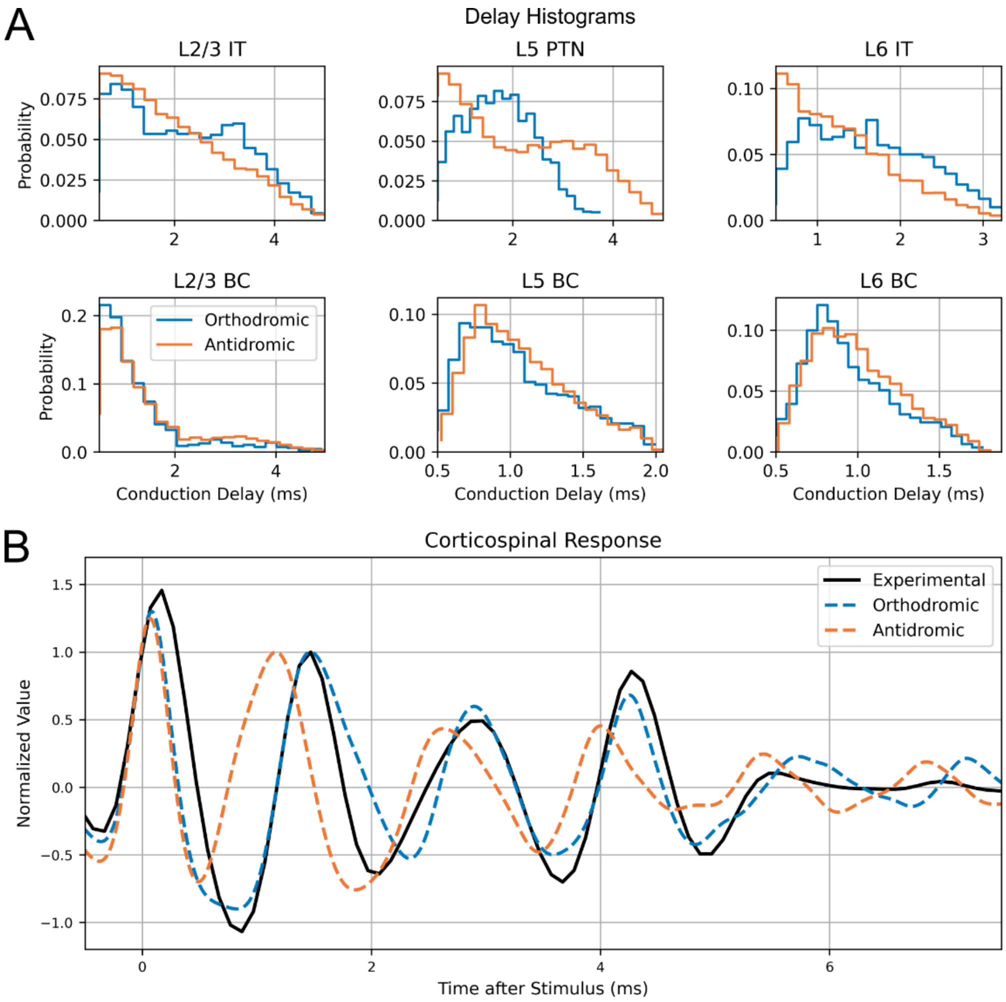

RFig. 1. *Histograms of conduction delay and simulated response due to activation methods. A) The resulting conduction delay histograms across all neurons of each cell type are plotted. B) The corticospinal response of the optimized model with each activation type is shown.*

Functionally with respect to conduction delays, orthodromic and antidromic activation result in similar histograms except for L5 PTN and L6 IT with the orthodromic-based delays exhibiting a later peak compared to antidromic-based delays (RFig. 1A). The effect on the corticospinal response in the model is that the I-waves are similarly proportionally delayed (RFig. 1B). The D-I1 interval is longer for the orthodromic method compared to the antidromic method and is consistent with the longer latencies in the L5 PTN conduction delay histogram. The key findings are that orthodromic vs antidromic activation result in a simple shift in the D-I1 interval that can be compensated for during optimization. Additionally, the difference in approaches have little effect on the population conduction delays for cell types other than L5 PTN and L6 IT.

To address this comment, RFig. 1 was added as a supplementary figure (S1 Appendix Fig G) in addition to the following in the main text:

Lines 566-572
"Though studies have demonstrated that motor thresholds in response to TMS are lower in presynaptic terminals [29], other studies have showed the primary axon to have a large influence on the sensitivity of a cortical pyramidal cells to TMS [48]. We found that antidromically propagated action potentials result in conduction delays that are similar to orthodromically propagated action potentials (S1 Appendix Fig G). Because this study used implicit, functional representations of axons through conduction delays, we found this strategy for activation to be representative of the current theories of activation."

3) In the Esser model, L6 inputs to L5 are relatively weak compared with those from L2/L3, which I think is correct physiologically. As far as I can see this is not the case in the present model. Perhaps as a result of this, input from L6 plays an important role in production of I2 and I3 waves. Previous physiological models have generally suggested that input from L2/3 would play a more important role. Could the authors comment?

In the original results, the optimized model L2/3 IT to L5 PTN projection had a lower synaptic strength than the L6 IT to L5 PTN projection. However, the sensitivity analysis revealed that L2/3 IT to L5 PTN input had a much stronger effect size than the L6 IT to L5 PTN input, which highlights one drawback of optimization without regularization. Parameters that do not strongly affect the objectives are free to take a larger range of values. Regularization adds penalties to large parameter values, and these penalties reduce parameters that do not affect the constraints, e.g. I-wave peaks. Upon closer inspection, several other large-value, low-effect parameters were identified in the model, and the regularization step was added to address all such cases.

Without adding any other constraints to bias the results toward these model values, the new model has a stronger L2/3 IT to L5 PTN connection than L6 IT to L5 PTN projection (RFig. 2) as expected by the Reviewer and in agreement with the literature [6,7]. Additionally, connections originating from deeper neurons have a lower connection strength to L5 PTNs. We created a new visualization of the final model parameters that allows easier interpretations of how the synaptic strengths relate to each other. This figure replaces S1 Appendix Fig A.

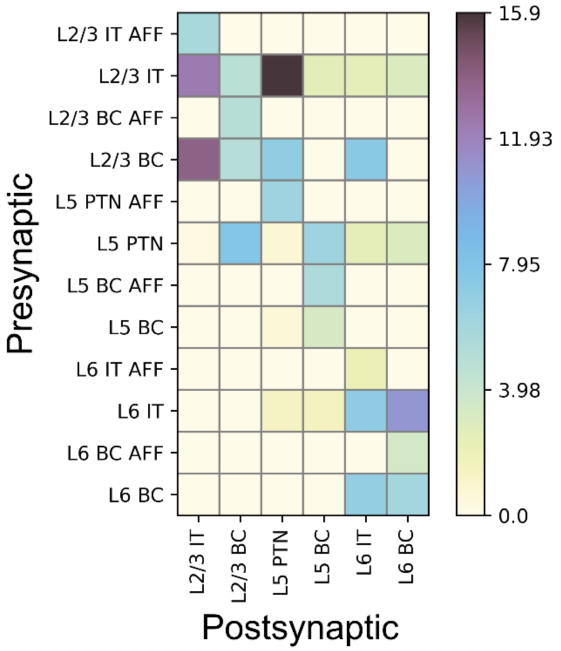

RFig. 2. *Maximal synaptic conductances of the optimized model. Presynaptic neurons are arranged on the y-axis, and their maximal synaptic conductance on their postsynaptic targets along the x-axis are coded according to the color bar.*

4) Fig A suggests that L6BC show greater activation in the D+ model than the D- model. Given that these synaptic inputs probably arrive too late to influence D-waves, why is this the case? I think its necessary not only for a computational model to work but also that we understand why it works.

In general, the differences in activation for the individually optimized models are not related to affecting the D-wave but rather to shape the subsequent activations that occur to compensate for the presence or lack of the D-wave. Triggering a D-wave will propagate activity from L5 PTNs back to other L5 PTNs. Activations for other neuron types, including inhibitory neurons, primarily affected the I-waves. The effect sizes (Fig. 3) show that the preferential activation of the D-wave is only achieved by activation of L5 PTNs.

We address this question with the following text in the Discussion:

Lines 293-298
"The unified model reproduced responses that included or excluded a D-wave primarily by changing the direct activation of L5 PTNs, which is consistent with the mechanisms of D-wave generation [4]. Other differences in activation (S1 Appendix Fig A) to generate a D+ or D-response were necessary to compensate for the transsynaptic effects on subsequent I-waves that were caused by a D-wave or reproduce the desired I-wave amplitudes without a D-wave."

5) Similarly, why does the L2/3 BC AFF – L2/3 BC play a role in I1-wave generation (Fig 6)

given that its contribution should arrive too late to influence the main monosynaptic input from L5 AFFs?

To clarify the labels in Fig. 6, the nomenclature of the variables is such that L2/3 BC AFF – L2/3 BC refers to the synaptic strength of the projection, not its activation via TMS. Changing the strength of a projection not only affects the evoked response but also the steady-state of the network model prior to receiving the TMS pulse. The change in the steady-state also changes the network's response to activation.

We found that reproducing the evoked response in addition to maintaining the appropriate steady-state firing rates proved to be opposed to each other during optimization (S1 Appendix Fig. E). During the two-variable-at-a-time analysis, the projection strengths changed both the steady-state firing and the evoked response. Only the activation parameters could specifically affect the evoked response without affecting the network state.

We added the following text to the Discussion to address this comment:

Lines 298-302
"The synaptic strength of intracortical projections had multiple effects on the model, including changing the steady-state properties of the network prior to receiving the TMS stimuli. Few projections could preferentially affect single corticospinal waves (Fig 7), and most affected multiple corticospinal waves due to their effect on the network state (S1 Appendix Fig E)."

6) For interest, a paper by Edgley et al (DOI: 10.1093/brain/120.5.839) notes that motor cortical PTN neurons may fire multiple times in response to a TMS pulse, which seems to be in contrast to the model predictions here.

Thank you for bringing this paper to our attention. Despite the individual traces that were used in most of the figures showing multiple spikes in response to TMS, the aggregate of all TMS responses is summarized in a separate figure (reproduced below) and supports single spike responses. The figure plots the histogram of the latencies of the action potentials evoked by TMS across all trials from single axons. The histograms do demonstrate evidence of multiple spikes with multiple peaks in the data. However, each histogram has one prominent peak around which most of the spikes are clustered, indicating that single spikes are the predominant response. This agrees with our model predictions that corticospinal tract neurons are more likely to respond with single action potentials.

We appreciate this comment as it brings to our attention a deficit in our communication of those results. A subset of corticospinal tract neurons in our simulations do respond with multiple spikes, but the majority responds with one spike. (~18% are silent, ~45% single spike, ~25% two spikes, ~10% three spikes, ~2% four spikes, S1 Appendix Fig. H). To address this, we promoted S1 Appendix Fig. H to be a main figure Fig. 9 in the Discussion.

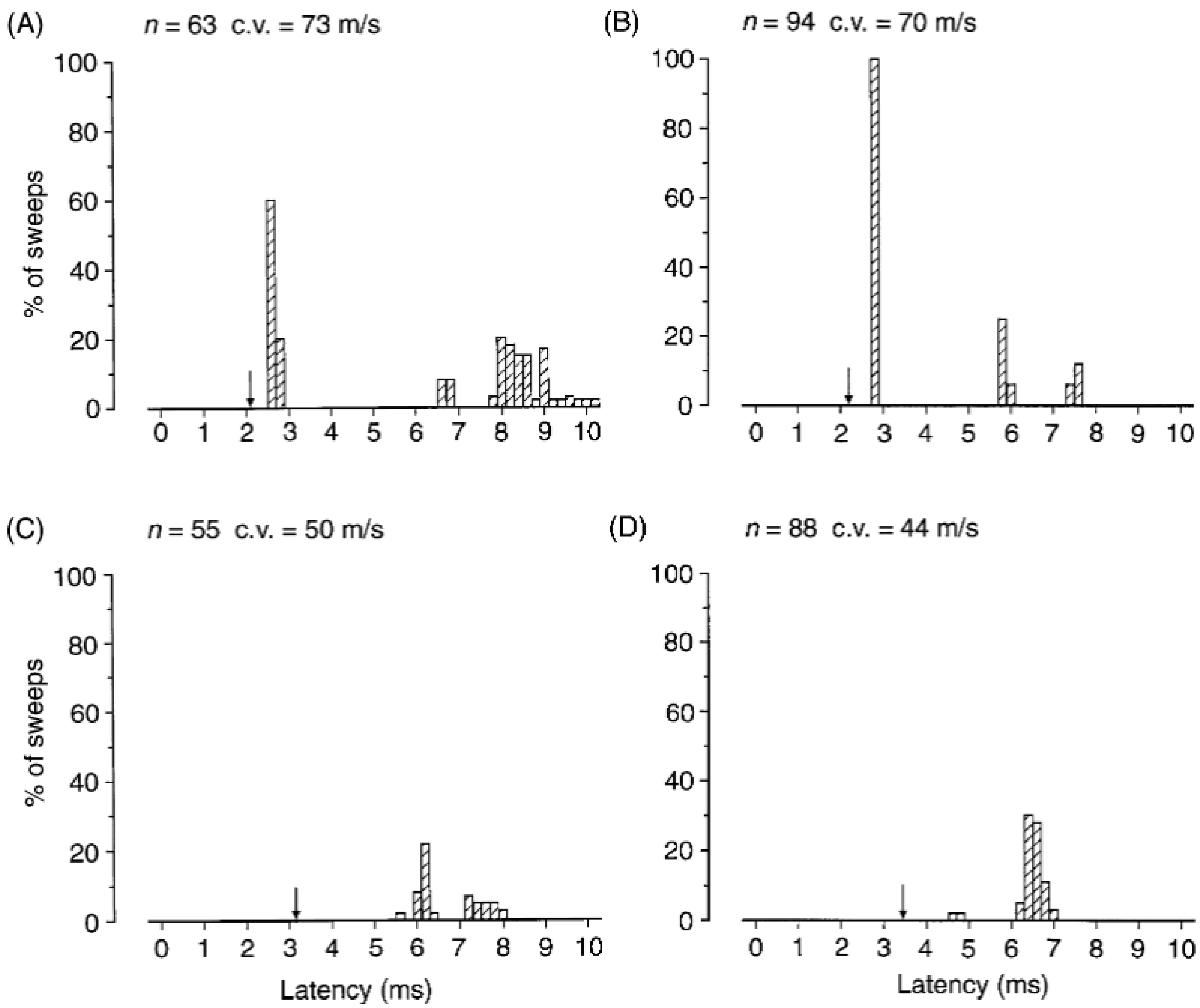

Fig. 2 Frequency distribution of responses to TMS in four axons at different latencies. The conduction velocities (c.v.) of the axons are indicated. Arrow indicates response latency to stimulation of the pyramidal tract.

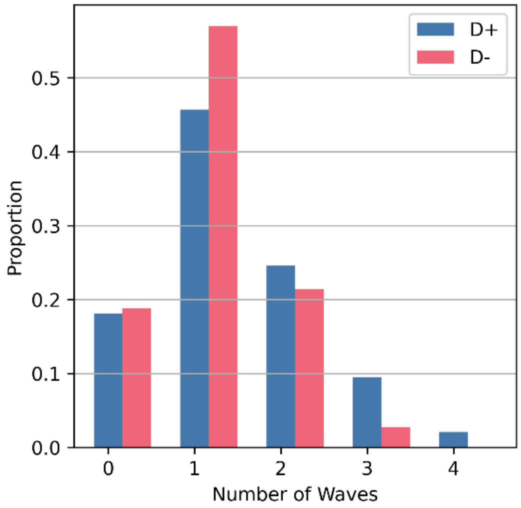

Fig 9. Histogram of number of waves for which L5 PTNs contributed a spike.
For each stimulus presentation, the spikes generated by each L5 PTN were divided based on the time windows for each corticospinal wave, and the total number of time windows during which spiking occurred was counted.

Reviewer #2

In the manuscript entitled: "Circuits and mechanisms for TMS-induced corticospinal waves: Connecting sensitivity analysis to the network graph," the authors presented a detailed study of the mechanisms underlying D- and I-wave generation and motor cortical stimulation via TMS. The methodological approach to investigate the mechanisms is built on a well founded model structure and includes optimization against real patient data, sensitivity analysis, and structural analysis. Studying the model behavior across a wide range of well defined parameters and constraints was a challenging task which the authors approached well. The study was also very well visualized and presented in the manuscript. In order to further improve the paper, I have the following suggestions:

Major comments:

#133: The Results section contains a lot of methods. I strongly suggest to leave out methods details in results, or refer to the methods supplying minimal detail in results. e.g how is the tms defined, input-output approach (just one example: #192-199)

We edited the Results to describe more concisely the methods by doing the following.

- The description of how the unified model was identified was moved to the Methods section.

- The paragraph #192-197 was deleted except a one-sentence introduction of two-variable-at-a-time analysis to provide context for readers who are unaware of the general technique.

#174: Whole section on unified models needs to be clarified. It is unclear where a weighted combination of parameters is taken, or parameters are identical, or the best individual parameters from one model are taken. Whenever the 'models' are referred to it is not exactly clear what is meant. Also 'unified D- model' and 'unified D+ model' are confusing #186, #187. Reconsider notation for each model.

We agree with the Reviewer that the numbers of models and references to these models is confusing.

It is first important to emphasize that the sensitivity analysis and structural analysis were conducted only on the unified model. The identification of the unified model parameters relied on interpolating between individually-optimized, subject-specific models. Because the description of the search for the unified model is not critical to the subsequent analyses, the description of this process and figures pertaining to the subject-specific models were moved to the Methods. The Results now contains only figures and descriptions concerning the unified model. We further refer to these models using their appropriate descriptors, unified vs subject-specific models.

#201: a sensitivity analysis of such a complex problem is usually conducted in close vicinity around the working point. Analyzing the sensitivity in the entire space is potentially not telling because it shows the global behavior including a lot of cases far from reality. These unrealistic cases have a big influence on the final sensitivities. When decreasing the range around the working point, the polynomial fits will get way better and the sensitivity coefficients will be more accurate. If possible, it would be very interesting to analyze the sensitivities in a smaller range and to compare them with the ones presented in the current manuscript.

We agree with the Reviewer that it is important to restrict the parameter range of the sensitivity analysis, but we find that the parameters are appropriately bounded and that a global sensitivity analysis is more aligned with the goals of our analysis than a local sensitivity analysis.

Spanning from 0 to 100% activation across all neurons would be informative, so we do not want to change the bounds for those parameters.

The upper bound for synaptic strengths was 10x the original model value and was selected to compensate for the reduced size of our model.

Reducing the range might also eliminate zero, an important value for certain parameters. Zero is crucial to include in the sensitivity analysis because a parameter's absence can reveal important information about its contribution to the system. Below (RFig. 3), we see that eliminating zero or

restricting the parameter ranges could place the sensitivity analysis in regions where the I-wave amplitude has little or no-change, resulting in a drastically reduced effect size. Thus, conducting the sensitivity analysis across this full-range, representing a more global sensitivity analysis, accurately characterizes the current model. A smaller range would be important to assess the robustness of the model solution, i.e., whether a local minimum in the parameter space was identified.

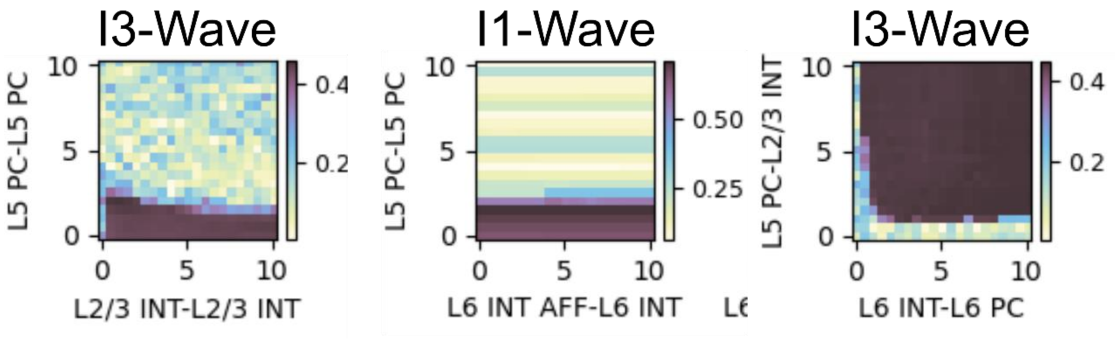

RFig. 3. *Surfaces measured from TVAT analysis. Colors represent the amplitudes of the labeled wave.*

We summarized this reasoning to define the boundaries of the TVAT analysis with the following text in the Methods:

Lines 802-808
"The TVAT analysis investigated the effect of activation parameters and projection strengths on corticospinal wave amplitudes. Activation parameters were varied between 0 and 1, representing no activation to full activation of a population. Synaptic scalars were varied between 0 and 10, representing a lesion of a projection to 10x the strength of the original source model. The 10x upper bound was chosen to compensate for the reduction in model size compared to the original source model. A global sensitivity analysis was chosen over a local sensitivity analysis that may be restricted only to evaluating the robustness of the model rather than be representative of a full characterization of the system."

Fig. 8: From these observations, it seems that the results did not converge yet with the selected time step of 0.025 ms (see number of spikes, ISI, etc.). From the convergence results you show, a step size of 0.01 ms would be more appropriate to ensure stable results while not being completely infeasible to realize I suppose. It leaves a discomforting feeling when interpreting the results. Please show that the final results, i.e. sensitivities etc., are not affected by the chosen stepsize.

We recognize the concerns in stability convergence. The initial 0.025 ms time-step was chosen using a knee-finding algorithm as a trade-off between accuracy and computational performance. However, we re-ran the simulations using a 0.01 ms time-step despite the increased execution times.

#327-328, #364-368: Please relate these important findings to the different hypotheses discussed in Ziemann (2020) to shed more light onto the potential mechanisms of I wave generation:
Ziemann, U. (2020). I-waves in motor cortex revisited. Experimental brain research, 238(7), 1601-1610.

We agree with the Reviewer that using these hypotheses to frame the results is a great way to organize the Discussion, and the following text was added:

Lines 316-334
"Several hypotheses have been proposed to explain I-wave generation and can be grouped into mechanisms at the network level vs the single neuron level [6]. Network level hypotheses propose either a single pathway of activation that simultaneously recruits multiple excitatory neuron populations to produce I-waves or multiple pathways of activation that recruit different excitatory neuron populations to produce different I-waves. Both hypotheses have a second variant that includes inhibitory neurons. The sensitivity analysis supports the hypothesis that multiple pathways are recruited to produce different I-waves and that interneurons serve an important role.

These network level hypotheses focused on I-wave generation being driven by afferents to the motor cortex, e.g., cortico-cortical fibers originating in premotor or somatosensory areas or projections from thalamus. However, in addition to supporting the large effect sizes of afferents on I-waves, the sensitivity analysis identified mechanisms of I-wave generation that were endogenous to the motor cortical circuit, i.e., activation of intracortical motor cortex projections can generate I-waves. This is a novel hypothesis produced by the computational analysis.

At the single neuron level, there are two major hypotheses. One is the concept of L5 PTNs as neural oscillators that burst during activation to produce I-waves. Our results do not support this hypothesis as the L5 PTN models tended to fire a single time during the course of the I-waves. These simulation results are consistent with recordings of single corticospinal axons in response to TMS [13]. The second hypothesis involves a mechanism involving calcium and the backpropagating action potential, but this hypothesis cannot be evaluated using our framework because the neuron models lack dendrites."

#435: The Limitations Section needs to be extended:

We agree with the Reviewer that these are important limitations to the work. We added the following text to address the bullet points.

- mention that the model is not capable of describing effects of directional sensitivity because E-field coupling is simplified (effects TMS coil orientation, PA vs AP etc.)
the D+ and D- experimental results come from different subjects, and it's not discussed how experimentally one can tune D-wave activation. (from recruitment/dose dependence and TMS coil orientation)

- there is no time dynamics to the stimulation (biphasic vs monophasic pulses), please discuss how your definition of current pulses and TMS activation could be generalized to specific waveforms (or not).

The following text addresses the previous two bullet points:

Lines 453-466
"However, point neuron representations precluded any analyses involving dendrites, axons, spatial integration of postsynaptic potentials, or ephaptic coupling. Spatially extended, i.e., morphologically realistic, neuron models, could accommodate these mechanisms and enable the exploration of their contributions to modulation of I-waves. Furthermore, this work represented TMS stimulation using an input–output approach, i.e., a given stimulus intensity resulted in some proportion of neurons of a particular type to fire an action potential. Therefore, the results cannot be generalized to explain effects to conditions beyond the experimental data it was optimized to reproduce, i.e., the results are only valid for coil orientations that induce an electric field in the posterior-anterior direction using a monophasic pulse. Without additional data, the results and model cannot be reliably extrapolated to responses in other orientations such as lateral-medial, for other stimulation waveforms such as biphasic pulse responses, or to generate I-waves beyond I3. More realistic representations of the electric field coupling could allow generalizations to other conditions by modeling the spatial distribution of activation of the induced electric field using finite element modeling [22,34–36]. Combining these methods with neuronal models with realistic morphologies [27–29] would yield informative insights."

- regarding dosage dependence (which you already mention) you could add that this kind of sensitivity analysis you did has to be actually done for every stimulation intensity because the mechanisms will change across the IO curve. If done, you could show a shift of mechanisms across intensities.

Lines 485-492
"The analysis could also be improved by including more types of data. Experimental data from only two subjects was used with responses from a single TMS intensity. The data were representative of the two qualitative types of responses—with and without D-wave. The small dataset allowed for more rapid model development due to fewer optimization constraints, and the methods established in this work can be applied in the future to extended data from more subjects and more recordings within subject. Furthermore, the optimization included only a single stimulus intensity as a constraint. Incorporating corticospinal recordings in response to multiple stimulus intensities from the same subject could reveal differences in effect size or engaged mechanism as a function of intensity."

#481: Conclusion Section is very vague, and non-specific to the detailed conclusions drawn throughout the results section. Please extend and be more specific.

We added the following sections to the Conclusion to describe more explicitly the findings of the study.

Lines 506-525
"To understand the mechanisms and principles underlying a biological process, sensitivity analysis is a powerful tool. However, as the number of relevant variables increases, the analysis can become overwhelming, and conclusions become diluted. At these large numbers, degeneracy in the sensitivity analysis is possible as many mechanisms can be identified to be significant to the phenomenon of interest. However, there is also the possibility that subsets of these mechanisms share certain properties that represent a more fundamental mechanism or at least a lower-level mechanism that was previously unclear or unaccounted for. In this case, a secondary analysis can reveal these lower-level mechanisms that underly the variables that explain the phenomenon of interest.

In this work, the sensitivity analysis supported one of the major hypotheses concerning I-wave generation: I-waves are recruited transsynaptically through separate circuits that impinge onto L5 PTNs and involve both excitatory and inhibitory neurons. Additionally, activation of afferents onto L5 PTNs and non-L5 ITs cells was important for I-wave generation. The secondary analysis revealed that the anatomical structure of the network, i.e., the wiring diagram and conduction latencies that resulted from the anatomical constraints, were then important for predicting the circuit activations that give rise to specific I-waves with both the recruitment of afferents to L2/3 and L6 IT cells being possible mechanisms.

Finally, the lower-level nature of the mechanism identified using the secondary analysis allows these insights to be generalized beyond the motor cortex and TMS. Understanding the circuit organization of the target neural system and its inherent conduction latencies can be used to screen for important pathways that are recruited and contribute to an acute evoked response."

#775: The Training Classifiers section is written quite abstractly, making it difficult to directly relate the machine learning methods to the details of your model. For example:

#784: Please define "class" and "samples" related to your model.

The classes during classification were defined using the following text. References to "samples" in this section were replaced with "data".

Lines 868-872
"Two types of classifiers were used based on the number of classes that needed to be identified by the task. Logistic regression was used for binary classification to identify whether an activation had a preferential or non-preferential effect on any corticospinal wave. Support vector classification (SVC) with a radial basis function was used for multiclass classification to identify the corticospinal wave on which a preferential activation had the greatest effect, i.e., the D-wave, I1-wave, I2-wave, or I3-wave."

#789: Please define your quantity of interest how you define true positive and true negative you validate against.

The description modified to clarify how accuracy was computed.

Lines 886-887
"Classification performance was quantified on the validation sets using accuracy, i.e., the proportion of classifications that were correct..

#806 Table 5: The definitions of the graph metrics are quite difficult to understand. Some would benefit from different wording, or potentially using mathematical notation

We addressed this by adding a description in the text and adding the mathematical equations in the table.

Lines 854-860
"Centrality attempts to quantify the importance of a node with different centrality metrics using different criterion. Closeness centrality computes the reciprocal of the average length of the shortest path between a node and all other nodes. Nodes with a higher closeness centrality are "closer" to all other nodes, and their dynamics can propagate more quickly throughout the graph. Harmonic centrality is the average of the inverse of the shortest path between a node and all other nodes, and characterizes sparse networks with greater sensitivity than closeness centrality [56]. Betweenness centrality measures the proportion that a node was included as a part of the shortest path between nodes [57]."

Table 5

Closeness Centrality [56]	Reciprocal of the average distance of the shortest paths between the node and all other nodes. A larger closeness centrality means that the node is closer to other nodes. $$C_v = \frac{N-1}{\sum_u d(u,v)}$$ where $d(u,v)$ *is the shortest path between nodes* u *and* v
Harmonic Centrality [56]	Sum of the reciprocal of the shortest path distances between the node and all other nodes. A larger harmonic centrality also indicates that the node is closer to other nodes. $$H_v = \sum_{u \mid u \neq v} \frac{1}{d(u,v)}$$ where $d(u,v)$ is the shortest path between nodes u and v
Betweenness Centrality [57]	Ratio indicating the proportion that a node is included in the shortest path between nodes.

$$B_v = \sum_{s \neq v \neq t \in V} \frac{\sigma_{st}(v)}{\sigma_{st}}$$

where σ_{st} is the total number of shortest paths from node s to node t and $\sigma_{st}(v)$ is the number of those paths that pass through node v.

Minor comments:

Clarify the difference between the machine learning model (sometimes called a neural network in some applications) and the point neuron network (aka Esser), to be sure that the distinction is clear between the machine learning methods deployed and the computational model developed.

To disambiguate the two methods, the machine learning model was referred to as a classifier, and the point neuron network was referred to as a neuronal network model.

#88 - 94: - clarify the definitions of each hypothesis

We chose to articulate the hypotheses described in Ziemann (2020) in the Discussion section.

#100 - 104: - rephrase talking about constraints to recordings. Constraint to recordings is not necessarily an advantage rather a different approach that works forward from first principles to DI-waves rather than fitting/exploring parameters strictly constrained to experiment.

To be consistent with the optimization nomenclature we changed references to "constraints" with "objectives". These changes were made accordingly across all instances.

Lines 103-105

"To determine the TMS activations and neuron-to-neuron projections that contribute to I-waves, we used experimental recordings of the corticospinal response to TMS to provide objectives to optimize a computational model of a motor cortical macrocolumn."

#165 Fig 4 caption, 'unified D– model' is confusing.

We simplified all references to a D+ or D- unified model as just the unified model.

#179 - 180: Stick to one notation for (D+ and D-) or (D-wave and non-D-wave) model

We edited the text to refer to the different response types as D+ and D- after the initial description of the abbreviations.

#445: Please motivate the choice for the factor of 1800. I suppose it is something like the

average number of segments in a compartment model but it comes a bit out of the blue here.

We added the following text in the Model Limitations and Future Directions Section that contains this statement.

Lines 444-452
“An important design criterion for the modeling work was computational efficiency to enable the parameter explorations necessary for optimization and sensitivity analysis. In general, computational gains came at the expense of biological details and constraints. However, the simplified model enabled more specific and in-depth computational analyses. To benchmark the difference, a Blue Brain L5 neuron model [27] with realistic dendrites was compared to the Esser L5 PTN point model. Both were driven by identical 1000 Hz Poisson spike trains with synaptic weights adjusted to produce identical output firing rates (5 Hz). Simulating 10 s of time resulted in execution times of 1500 s for the Blue Brain model and 0.8 s for the Esser model. Hodgkin-Huxley style models with realistic morphologies could be up to 1900x slower than leaky-integrate-and-fire models.”

#516: ‘matched the range of microcolumns per macrocolumn’ may be more clear with ‘range’ replaced by ‘ratio.’

We made this replacement on lines 546-548.

“The macrocolumn was comprised of microcolumns that were arranged in a triangular lattice with a spacing of 50 µm [44] resulting in 79 microcolumns and matched the ratio of microcolumns per macrocolumn [42,45].”

#540 - 542: Could be rephrased for clarity. Based on the specified activated proportion from stimulus for the chosen afferent or neuron type, a corresponding proportion of that given population or afferent type was randomly chosen to be presented with a stimulus.

We updated the text with this suggestion on lines 559-562.

“TMS activation included only suprathreshold effects. Based on the specified activated proportion for the chosen afferent or neuron type, a corresponding proportion of that given population or afferent type was randomly chosen to be activated, and neurons/afferents were randomly selected for each presentation of the stimulus.”

#543: Please provide information about the current pulse, i.e. pulse duration and amplitude.

The following text was modified to include information about the stimulus:

Pulse duration
Lines 642-644
"Monophasic pulses were delivered with a Magstim 200^2 stimulator (The Magstim Company Ltd., Whitland, UK), once every 5 seconds. Pulses had a rise time of 100 µs and a duration of 1 ms."

Pulse amplitudes were reported in terms of their individual resting motor thresholds and the maximum stimulator output.
Lines 645-649
"Two subjects were included in this study (Fig 1B) with the stimulator output set to 120% of their respective resting motor thresholds (RMT). Subject 1 was female, 64 years old, and had a cervical epidural electrode implanted at C3–C5 level; the RMT of TMS was 34% of maximum stimulator output. Subject 2 was male, 68 years old, and had a cervical epidural electrode implanted at C1–C2 level; the RMT was 55% of maximum stimulator output."

#548 - 549: Reading that connectivity rules for each microcolumn are identical, it is now unclear to me whether connections are only formed within microcolumns or also across them.

We want to clarify that in our model, neurons form connections within and across microcolumns. Regarding the specific lines referenced above, we rephrased the consequence of removing orientation selectivity-based connectivity.

Lines 573-578
"Connectivity parameters, neuron parameters, and synaptic parameters were identical to those reported in [7] with the following exceptions. Orientation selectivity-based connectivity was not included, so microcolumns could connect to any of their neighbors rather than being restricted to microcolumns with similar orientation sensitivity. Because the geometric area of the model was reduced from the original, the overall synaptic drive was decreased, and the subsequent optimization allowed larger synaptic weights to compensate."

#553: I suggest to reformulate this sentence (measurements -> observations, simulations, or stimulations)

We made the following changes.

Lines 580-583
"Simulations were designed to ensure that the network achieved steady-state before firing rates were measured, and steady-state properties were measured between 500 and 2000 ms.. To reduce synchronization of the network due to simultaneous activation of afferent inputs, the onsets of the Poisson spike trains of the afferents were randomly and uniformly selected between 0 and 200 ms."

#565: Is 20 seconds the actual time it takes to run the model once given some PC hardware? If so please provide details about the PC hardware. Or is it the simulated time

in the model to study the time steps in terms of diverging solutions? In #560 you write that the total simulated time is 3000 ms in the model. Please be more clear here and try to reformulate "simulation time" in the real world to run the model and "simulated time" inside the model. I'm confused. If not already done please also report the actual simulation time on the used PC hardware.

We updated the Methods with the following text to describe better the benchmarks of the simulations. We use "simulated time" to refer to the model time and "execution time" to represent clock time. Because most of these computations were run in parallel, the execution times are reported as compute-hours.

Lines 589-592
"Simulations and analyses were run on the Duke Compute Cluster on nodes comprised of a heterogeneous mix of hardware including the Intel(R) Xeon(R) CPU E5-2680 v4 @ 2.40GHz, Intel(R) Xeon(R) CPU E5-2680 v3 @ 2.50GHz, Intel(R) Xeon(R) Gold 6154 CPU @ 3.00GHz, Intel(R) Xeon(R) Gold 6254 CPU @ 3.10GHz."

Lines 584-588
"TMS stimuli were applied at 2000 ms with inter-trial intervals of 200 ms with a total of five trials. This interval was selected based on population averages of trials ,which showed no longer-term effects beyond 150 ms. Furthermore, the model did not include synaptic plasticity or thalamic connections. Analysis of the TMS response was conducted on the trial average. The total simulated time was 3000 ms and had an execution time of 49 s."

Lines 595-597
"The time-step was selected by running single neuron simulations while log-linearly varying the time-step from 0.001 to 0.2 ms. The models received a random Poisson input with a mean firing rate of 1000 Hz for 20 s of simulated time."

Lines 670-680
"Optimization used 499 particles and ran for 300 iterations before termination. The optimization was initially repeated for each model five times to increase coverage of the parameter space and the likelihood of locating a global best solution. A best model was then selected, and a subsequent regularized optimization was repeated five times to identify a regularized model. Optimization evaluated particles in parallel across 499 CPUs while using a single main CPU to collect, analyze, and update particle positions. Each iteration took an average of 444 s, which is greater than the execution time of a single simulation, but the communication overhead, file i/o, and analysis after each iteration of particle evaluation added extra time. Each optimization had an average execution time of 37 hours, or 18,500 compute-hours. The two subject-specific models had ten total optimization runs using a total of 370,000 compute-hours to complete. Optimizations utilized an average of 131.21 GB of RAM."

Lines 811-812

“TVAT simulations were parallelized across 1,000 CPUs with a total execution time of 2,639 compute-hours, using 960 GB of RAM.”

Lines 824-826
“Polynomial regressions of the TVAT surfaces were computed using a single CPU with an execution time of 3.6 compute-hours, using 270 MB of RAM.”

Lines 907-908
“Feature selection for logistic regression and SVC was parallelized across 250 CPUs. Their total execution times were 556 and 2,083 compute-hours, respectively, and both used 35 GB of RAM.”

#569: Please describe shortly how the van Rossum spike distance is defined.

The following description of the van Rossum spike distance was added:

Lines 604-610
“The van Rossum spike distance was computed by convolving two spike trains using a causal exponential kernel and computing their L2 norm following equation

$$spike\ distance = \sqrt{\frac{2}{\tau}\int_0^\infty [h(t;\ u) - h(t;\ v)]^2 dt}$$

where τ is the time constant of the causal exponential kernel, $^2/_\tau$ is a normalizing factor, *h(t)* is the kernel function, and *u* and *v* are the two spike trains. A time constant of 500 ms was used for the spike distance because the 0.001 ms time-step case had a mean ISI of approximately 500 ms.”

#577: in Fig. 8 it can be seen from “Number of Spikes that the knee is below 0.03 ms. Why was 0.03 chosen as the reference (see major comment before).

The 0.03 ms reference was computed using a knee-finding algorithm, which determined the point along the curve at which the decrease in error relative to the increase in time-step (and therefore increase in execution time) began to diminish.

However, we decided to complete the work using the 0.01 ms time-step to eliminate any concerns.

Fig 8: “Normalized” and “Difference” curves need more explanation in the main text. Please also indicate with a dashed (colored) line also the final step size you selected (0.025). Please label the y-axis and units of the Coefficient of Variation (ISI) mean plot (middle left)

We are removing the figures and text regarding the knee-finding algorithm as we selected the 0.01 ms time-step.

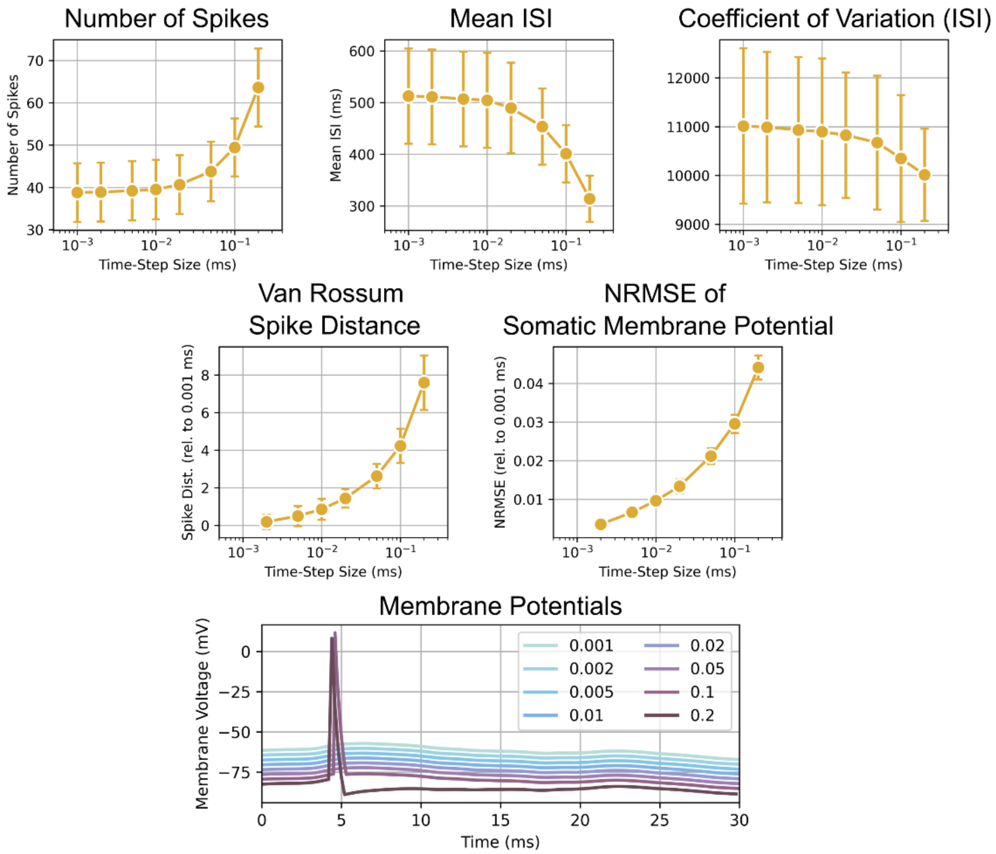

Fig 10. Analysis to select a time-step that both minimizes computation time and is numerically stable.

Each scatter-line plots shows the mean of a metric as a function of the time-step size in the log10 scale. At the bottom, the membrane potentials of the neuron model for different time-steps are shown. Offsets were added for the y-axis to allow all lines to be distinctly seen. The plots depict a key behavior that differentiates simulations at larger time steps. A pronounced afterhyperpolarization is seen with a 0.2 ms time-step that is absent from other time-steps. Additionally, spikes are generated at larger time-steps (0.1 and 0.2 ms) that are absent for smaller time-steps. These dynamics contribute to the larger numbers of spikes, lower mean ISIs, larger NRMSE, and larger spike distance observed for larger time-steps.

#608: Please report the specific coil type

The description of the coil is reproduced below:

Lines 639-642
"A figure-of-eight coil with external loop diameter of 70 mm was held over the right motor cortex at the location at which the threshold to elicit motor evoked potentials measured at the first dorsal interosseous (FDI) was with lowest, the induced current flowing in a posterior–anterior direction across the central sulcus."

#609: I suggest rephrasing “optimal scalp position” here because no one really knows where the optimal location really is.

Lines 639-642
We replaced “optimal scalp position” with “the location at which the threshold to elicit motor evoked potentials measured at the FDI was lowest.”

“A figure-of-eight coil with external loop diameter of 70 mm was held over the right motor cortex at the location at which the threshold to elicit motor evoked potentials measured at the first dorsal interosseous (FDI) was lowest, with the induced current flowing in a posterior–anterior direction across the central sulcus.”

#616-617: Is it not a concern that the D+ and D- cases are from different subjects? (--Aaron to Konstantin)

The intensity at which the D-wave is evoked is subject-dependent. For most subjects, D-waves do not appear until the stimulator output is increased greatly. For few subjects the D-wave is easily evoked at lower intensities. We wanted to understand the degree to which differences in electrical coupling of the induced field could explain these differences.

Our unified model suggests that the difference between these types of responders is due to differences in the distribution of activation, primarily whether direct activation of L5 PTNs was achieved. Practically this is likely caused by differences in the distribution of the induced field and the subject’s specific anatomy.

We have added the following text to address these concerns:

Lines 650-651
“These subjects were selected to investigate the mechanisms that underly these differences in the evoked response, given the same RMT intensity.”

#628: It is not clear to me what ‘best solution found by itself’ means. What is ‘it?’

“Itself” refers to the best solution found by the particle itself, and the text was updated to clarify this point.

Lines 664-668
“The particle’s position represents the parameter values for the model, and a velocity term updates the position using a weighted combination of the best solution found by the particle itself (cognitive best) and the best solution found among a particle’s neighbors (social best). PSO was implemented by modifying the *inspyred* Python software package [44].”

Fig 9: ‘Generation’ on the x-axis is not mentioned in the main text, and ‘iteration’ is used in the figure caption. Would be best to pick one term for consistency.

We replaced “Generation” with “Iteration” throughout the figures and text.

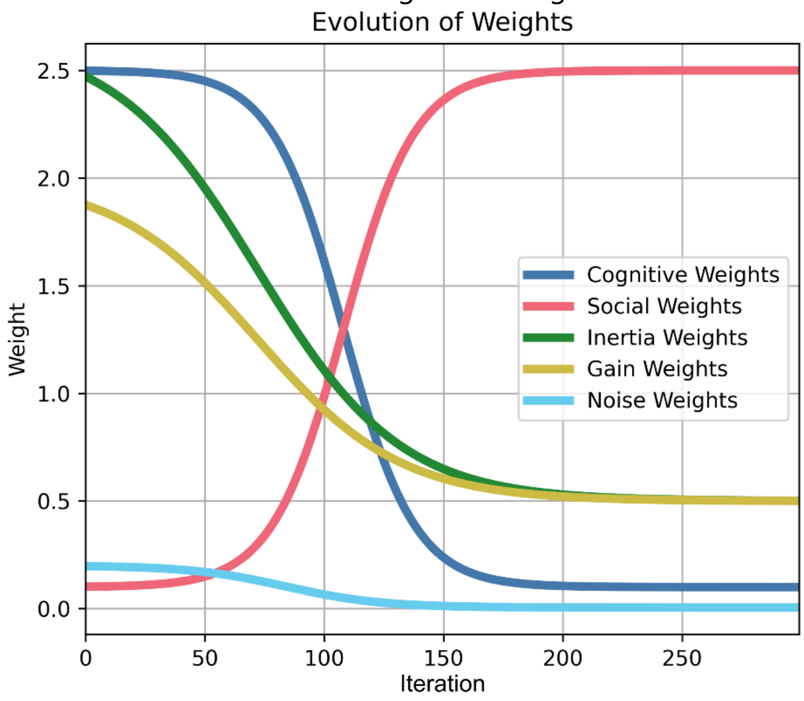

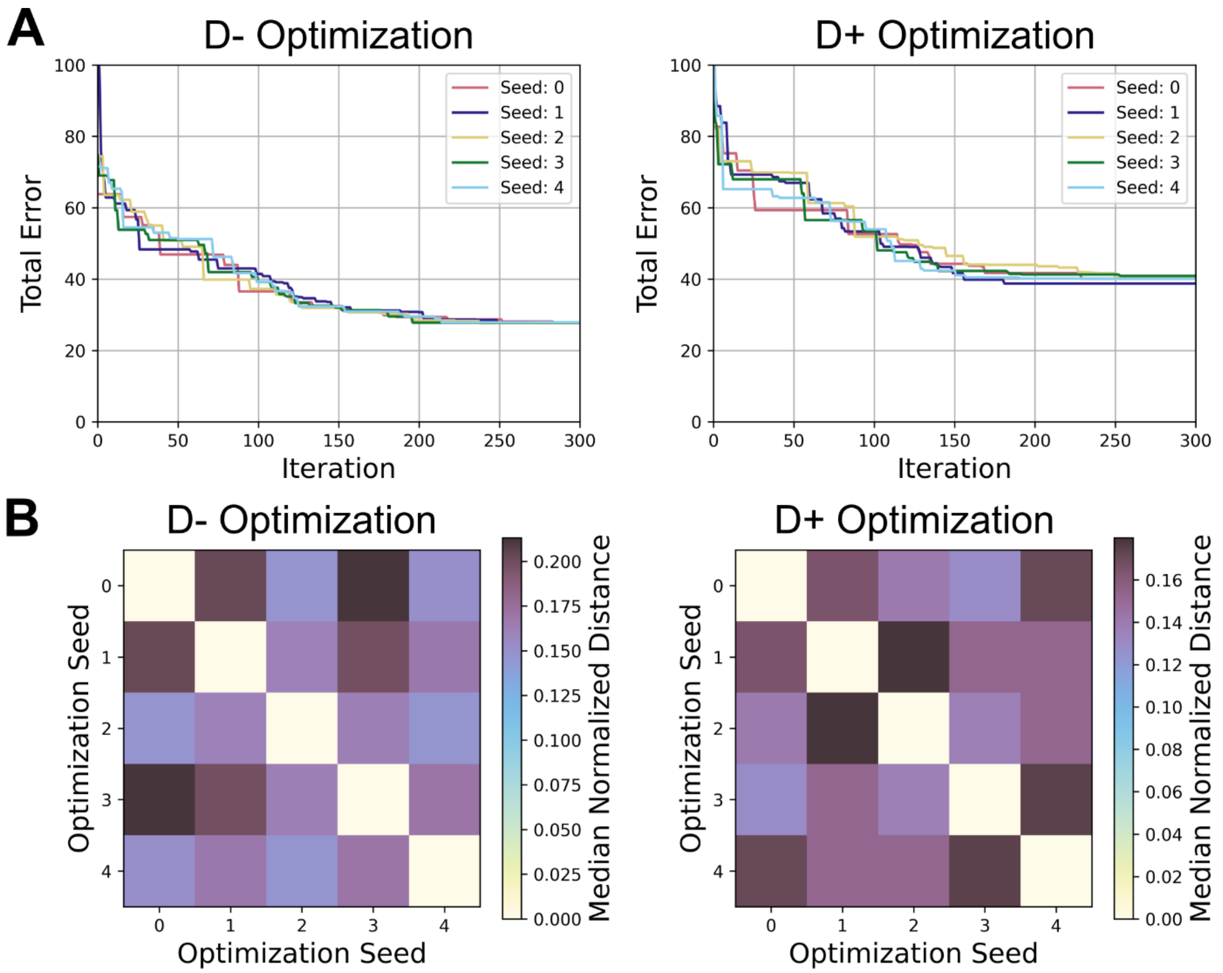

#655: 'optimization parameters' -> 'optimization hyperparameter' could clarify these are independent of the optimized parameter space.

We agree it is important to make this distinction and replaced references to the parameters determining the behavior of the optimization algorithm with "optimization hyperparameters". Previous references to "metaparameters" were also changed to "hyperparameters".

#669: Clarify the calculated difference here, unclear what this sentence means.

We added an equation to clarify how the boundary condition was implemented.

Lines 712-717
"A damped, reflecting boundary condition was implemented on the parameter search space [48]. If a coordinate of a particle's updated position exceeded its boundary, then the coordinate was reflected back into the valid parameter space using the difference between the original, non-valid coordinate and the boundary. Additionally, the reflection was damped by multiplying the difference with a scalar sampled from a uniform distribution between 0 and 1.

$$x_{reflect} = bound - U(0,1) * (x_{new} - bound)$$

"

#673: Please describe the constraint category "well-behaved" in a bit more detail.

The "well-behaved" category was a catch-all for objectives that didn't clearly fit into the other categories (corticospinal wave, firing rate, and synchrony). We recognize that "well-behaved" is an incorrect and misleading label for this category. To avoid confusion, we have renamed it to "miscellaneous".

Lines 720-723
"There were four main groups of objectives: baseline activity, TMS response, synchrony, and miscellaneous. The miscellaneous group included objectives that didn't fall into the previous groups, but were also not well-related to each other. However, this lumping was necessary to reduce the dimensionality of the pareto front for visualization."

Lines 749-753
"The miscellaneous group included the following objectives. A possible aberrant network behavior resulted in spiking activity of the network being dominated by large firing rates in a few neurons with the remaining neurons being silent. To avoid this, the standard deviation of the mean population ISI within a neuron type was minimized to prevent highly skewed distributions of activity. Another objective acted to identify the minimum noise added to the neurons."

#693 - 694: It may be prudent to mention somewhere that by only optimizing against recordings with 3 I-waves, the model is not trained to exhibit possible further bursts, and assumes only these 3 I-waves can exist.

We added the following text to the Discussion.

Lines 461-463
"Without additional data, the results and model cannot be reliably extrapolated to responses in other orientations such as lateral-medial, for other stimulation waveforms such as biphasic pulse responses, or to generate I-waves beyond I3."

#728: It sounds like you used a generalized polynomial chaos expansion of order 3 and extracted the Sobol indices from it (#742). Is this correct?

We cannot claim to have used a generalized polynomial chaos expansion because orthogonal polynomial basis functions were not used. Rather, we used polynomial regression with up to 3rd order polynomials and interaction terms. These polynomials (e.g., x, x^2, x^3) are not orthogonal.

Generalized Sobol indices were not used in the calculation of the effect size. Rather the absolute values of the coefficients from the polynomial regression were used as a surrogate for effect size.
This was clarified by adding the following text:

Lines 813-815
“The effect size for each parameter on a corticospinal wave amplitude was computed by fitting the surfaces generated by TVAT simulations via polynomial regression and summing the absolute values of the coefficients to represent the effect size. For each pair, the relationships between the two parameters and the amplitudes for each corticospinal wave were approximated using linear regression with elastic net regularization and a third-order polynomial model that included third-order interaction terms.”

Lines 827-830
“The partial effect size of a parameter for a corticospinal wave was represented as the sum of the absolute values of the coefficients of the polynomial models that involved the parameter. The total effect size for a corticospinal wave was calculated as the sum of the effect sizes across all polynomial models, i.e., across all pair-wise interactions, that included the parameter.”

#767 - 768: What are the divergence, convergence, and centrality measures calculated? Is this obvious? Ah ok this is mentioned in Table 5

Addressed in a previous comment.

Table 5: Some of these written descriptions are quite confusing. Especially Harmonic Centrality, which I needed to try and work out in summation notation to understand (maybe). It is possible that some of these descriptions would be better suited for mathematical/symbolic notation than as written descriptions.

Addressed in a previous comment.

Reviewer #3

The authors developed a point-neuron network model of the human motor cortical macrocolumn to simulate realistic D-waves and I-waves in response to single-pulse TMS. This model, based on a previous cortical model by Esser et al, allowed D-waves to be included or excluded by adjusting the activation of L5 PTNs. The 98 model parameters (related to synaptic weights, conduction delays, afferent delays, proportion of neuronal activations and noise) were optimised to achieve good performance for several constraints, including (asynchronous) baseline activity as well as TMS responses in the form of corticospinal waves (constrained by human single-pulse TMS data).

First, the authors performed a sensitivity analysis to identify key afferents and neuron

types within the motor cortex that generate corticospinal waves when activated. The sensitivity analysis showed that activation of the L5 PTNs mainly influenced D-waves, while afferents to the L5 PTNs significantly influenced the I1-wave. Direct activation of afferents was crucial for the generation of all I-waves, contradicting the idea that I-waves are generated by repetitive firing of individual neurons. Interestingly, the I3 wave was more sensitive to afferent input, the I1 wave was more sensitive to intracortical synaptic parameters, whereas the D wave showed no sensitivity to synaptic parameters. The waves were equally sensitive to excitatory and inhibitory neurons.

Secondly, the authors addressed the issue of degeneracy revealed by the sensitivity analysis (degeneracy in the sense of multiple different mechanisms contributing to cortico-spinal waves). To this end, the authors performed a structural analysis based on graph theory and machine learning (logistic regression with lasso regularisation, recursive feature elimination with support vector classification). The structural analysis focused on the wiring diagram and conduction latencies and helped to find common network features that contribute to cortico-spinal wave generation. Neuron types with high connectivity to L5 PTNs were found to contribute significantly to I-waves. High connection probabilities to L5 PTNs and the conduction delay of the shortest path to L5 PTNs were critical factors in determining the latency of an I-wave. Interestingly, inhibitory interneurons influenced several I-waves.

This is a solid and carefully conducted and interpreted computational study, using advanced methods to optimise parameters and estimate causal relationships between the many model features and the successful simulation of experimental results.

Major issues:

- Were the successful parameters (e.g. conduction velocities, afferent delays, conduction delays) within a plausible biological range? See also next question.

The conduction velocities remained in a biologically plausible range (RFig. 4). It is unknown what the afferent delays due to stimulation would be, but the optimization restricted the afferent delays to arrive at the postsynaptic somata within 3 ms of the stimulus.

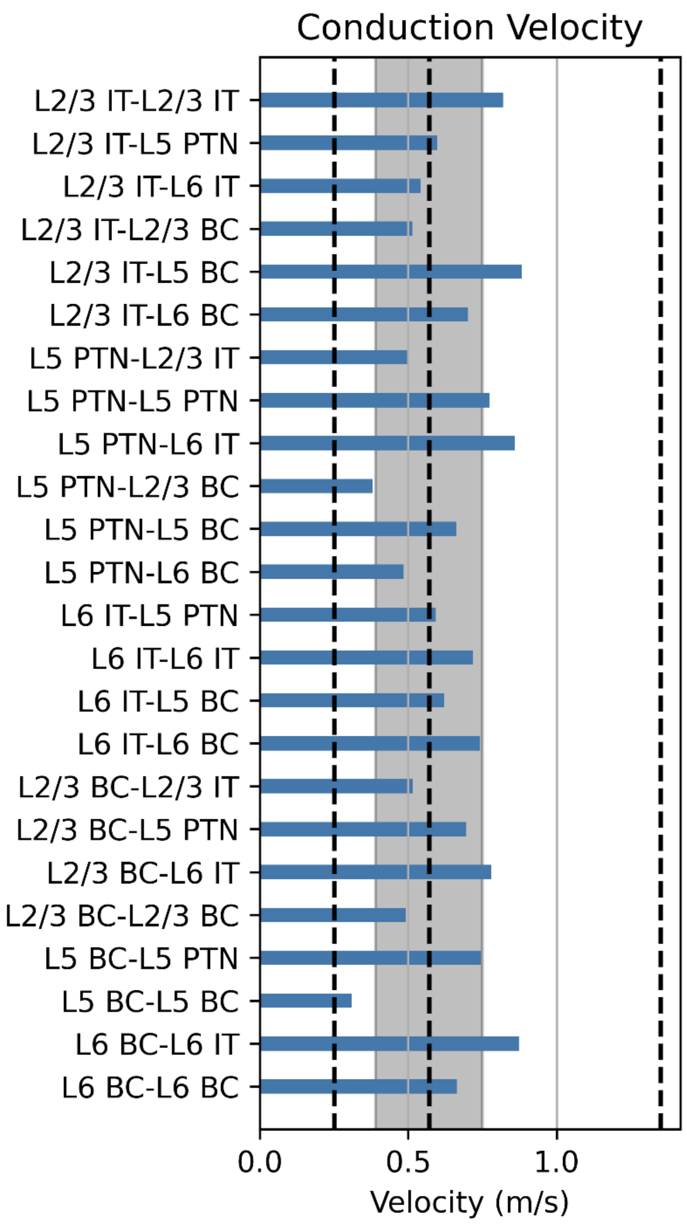

RFig. 4. *Final conduction velocities in optimized model. The left- and right-most dashed lines correspond to the total range of values reported in [41]. The middle-dashed line corresponds to the median value. The shaded region represents the median absolute deviation.*

This figure was used to replace the previous figure in S1 Appendix Fig B. Additionally, the following was added to the Discussion:

Lines 301-302
"Furthermore, the final conduction velocities of the model were within experimentally reported ranges (S1 Appendix Fig B)."

- Line 542: „Direct activation of neurons resulted in an injection of a short suprathreshold current to elicit an action potential that was propagated orthodromically to all postsynaptically connected neurons using all relevant conduction delays. Direct activation of the terminals of afferents resulted in the activation of all connected synapses with the appropriate conduction delays." Current theory and models of TMS (see e.g. Siebner et al. Clin Neurophysiol 2022) assume that spikes are triggered by TMS in the axon terminals (not in the somata). It seems that in your simulations of direct activation of neurons you assumed direct somatic activation and subsequent orthodromic spike propagation along the axon (simulated implicitly in the conduction delays). Can you comment on this? How would your results change if you assumed that TMS induces axonal spikes in the axonal tips (instead of somatic spikes that then propagate orthodromically), and if you shortened the conduction delay by omitting the orthodromic spike propagation? If this has a bearing on the interpretation of the results, you should include a short paragraph mentioning this.

We agree with the Reviewer, based on the results of simulation studies, that TMS activation of neurons occurs primarily in axon terminals, and we highlight details of two representative studies that motivated our implementation of activation. In short, these studies demonstrate that the main site of activation is likely the primary axon, but uncertainties remain whether the axon terminals branching from the axon collateral or the portion of the axon that enters and turns within the white matter are more important. To assess the functional consequences of activation in along the primary axon or at presynaptic boutons, we computed the histograms of the conduction delays that were produced using an orthodromic vs antidromic model. We found that for most cell types, the differences in conduction delays were negligible, and differences that did arise would be straightforward changes in the timings of the I-waves, that would be compensated for by the optimization process to reproduce the experimental data.

Salvador et al., 2011 [4] had pyramidal tract neuron models with main axons that extended into the white matter and simplified local axon collaterals. Aberra et al., 2020 [5] had anatomically realistic local axon collaterals and a main axon that extended primarily toward the white matter, but the main axon was truncated and did not enter the white matter. In [4], studies showed that L5 PTNs had lower thresholds in the main axon where it entered the grey matter and turned towards the internal capsule. In [5], the axon terminals had the lowest thresholds but the orientation threshold map had a significant component aligned with the main axon. When the main axon was removed, the orientation threshold map was significantly affected along the orientation aligned with where the main axon used to be. This effect was also consistent for L6 ITs. The importance of the main axon motivated the computation of L5 PTN conduction delays using the soma as a reference point, with uncertainty in its exact initiation point along its path to the white matter to be solved as an open parameter during optimization, i.e., the conduction velocity.

However, the prior interpretation does not apply to the remaining cell types. To characterize the functional difference between orthodromic vs antidromic activation, we computed the population conduction delay histograms for each approach. We labelled the original method as

orthodromic which used the cell body as the initiation point. We labelled the second method as antidromic, and in this approach randomly selected a single presynaptic terminal to be activated. The activation then propagated antidromically to activate all other terminals connected to the same axon. The histograms are shown in RFig. 1A.

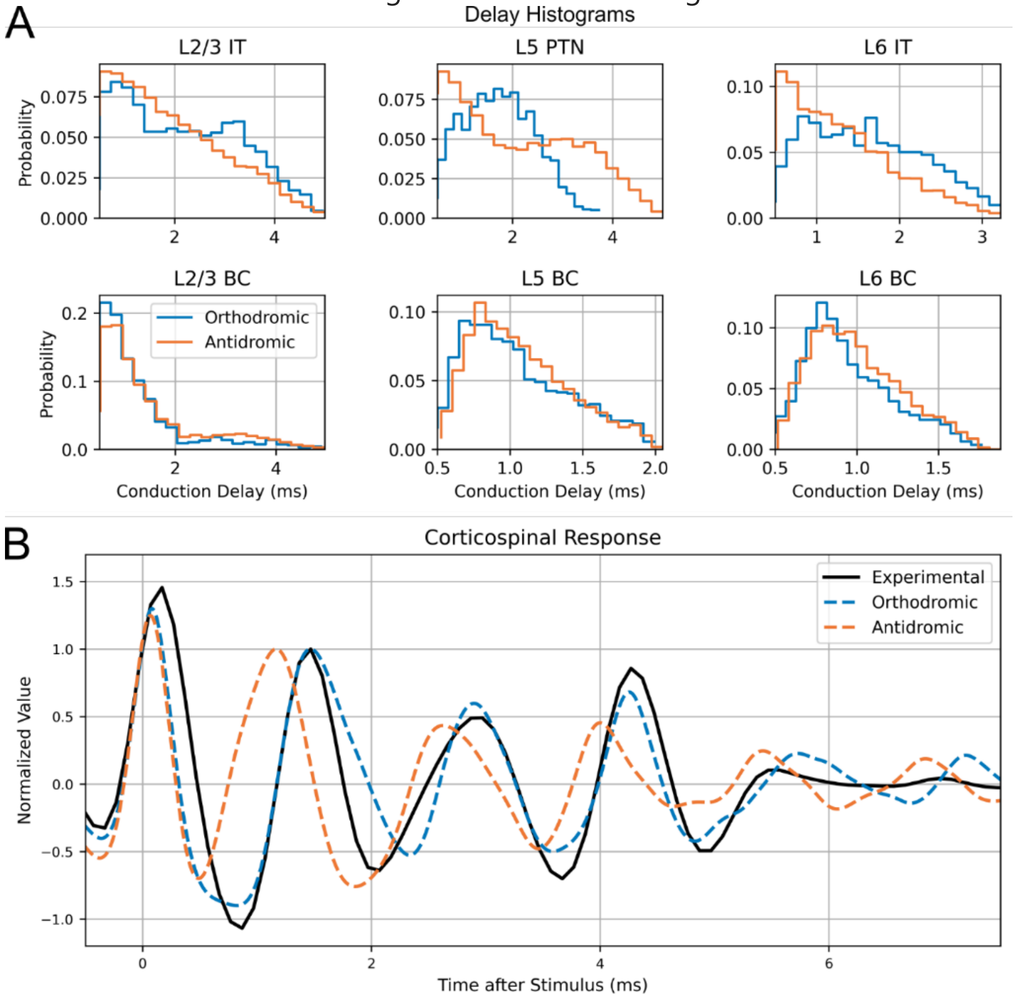

RFig. 4. *Histograms of conduction delay and simulated response due to activation methods. A) The resulting conduction delay histograms across all neurons of each cell type are plotted. B) The corticospinal response of the optimized model with each activation type is shown.*

Functionally with respect to conduction delays, orthodromic and antidromic activation result in similar histograms except for L5 PTN and L6 IT with the orthodromic-based delays exhibiting a later peak compared to antidromic-based delays (RFig. 1A). The effect on the corticospinal response in the model is that the I-waves are similarly proportionally delayed (RFig. 1B). The D-I1 interval is longer for the orthodromic method compared to the antidromic method and is consistent with the longer latencies in the L5 PTN conduction delay histogram. The key findings are that orthodromic vs antidromic activation result in a simple shift in the D-I1 interval that can be compensated for during optimization. Additionally, the difference in approaches have little effect on the population conduction delays for cell types other than L5 PTN and L6 IT.

To address this comment, RFig. 1 was added as a supplementary figure (S1 Appendix Fig G) in addition to the following in the main text:

Lines 566-572
“Though studies have demonstrated that motor thresholds in response to TMS are lower in presynaptic terminals [29], other studies have showed the primary axon to have a large influence on the sensitivity of a cortical pyramidal cells to TMS [48]. We found that antidromically propagated action potentials result in conduction delays that are similar to orthodromically propagated action potentials (S1 Appendix Fig G). Because this study used implicit, functional representations of axons through conduction delays, we found this strategy for activation to be representative of the current theories of activation.”

- The authors write (l. 725): „Generally, a solution that better matched the experimentally-recorded corticospinal waves had a worse match with the desired baseline activity.“ Can the authors briefly discuss in the paper how to improve this trade-off in the performance of the model or – if I have missed it - can they give me the line numbers where they have already done this?

To control independently the firing rate and to account for unknown sources of inputs, a separate form of suprathreshold noise was injected into each neuron. This is described in lines 536-539 of the revised manuscript:

“Noise was added to the neuron models that was independent of the synaptic drive provided by the afferents and unaffected by TMS to ensure proper baseline firing rates and reduce network synchronization. Each neuron received its own noise in the form of short, suprathreshold current injections with Poisson-distributed intervals.”

- The authors used a relatively old M1 point-neuron model (Esser et al. 2005). They had good reasons for doing so (ability to simulate I-waves), but as an outlook they could mention newer, much more realistic models that could be used in the future for TMS modelling - e.g. Bill Lytton's model (https://pubmed.ncbi.nlm.nih.gov/37300831/).

We agree that more realistic models are crucial for a more complete understanding of the system. We added text the Discussion to address this. Please see the next comment for the added text.

- The authors did not explicitly model the electric fields generated by TMS (see e.g. Mantell et al. Neuroimage 2023), but used direct activation of neurons and afferents as a proxy for TMS. A suggestion: in the discussion, the authors could briefly mention a possible future extension or combination of their network modelling with recently emerging multi-scale TMS modelling approaches (Aberra et al. J neural Eng 2018, Shirinpour et al. Brain Stimul. 2021, Weise et al. Imaging Neuroscience 2023), e.g. applied to more realistic M1 models (see above). It would be potentially very interesting to

combine such multi-scale TMS electric field modelling with anatomically highly detailed M1 models and with the authors' powerful sensitivity analysis and machine learning and graph theory based structural analysis.

We agree that more realistic neuron and electric field models will lead to more insights into the effects of TMS on cortex. We updated the Discussion with the following text to address these comments.

Lines 453-466
"However, point neuron representations precluded any analyses involving dendrites, axons, spatial integration of postsynaptic potentials, or ephaptic coupling. Spatially extended, i.e., morphologically realistic, neuron models, could accommodate these mechanisms and enable the exploration of their contributions to modulation of I-waves. Furthermore, this work represented TMS stimulation using an input–output approach, i.e., a given stimulus intensity resulted in some proportion of neurons of a particular type to fire an action potential. Therefore, the results cannot be generalized to explain effects to conditions beyond the experimental data it was optimized to reproduce, i.e., the results are only valid for coil orientations that induce an electric field in the posterior-anterior direction using a monophasic pulse. Without additional data, the results and model cannot be reliably extrapolated to responses in other orientations such as lateral-medial, for other stimulation waveforms such as biphasic pulse responses, or to generate I-waves beyond I3. More realistic representations of the electric field coupling could allow generalizations to other conditions by modeling the spatial distribution of activation of the induced electric field using finite element modeling [22,34–36]. Combining these methods with neuronal network models with realistic morphologies [27–29] would yield informative insights."

- Could the authors comment on the mechanistic explanations for the effects of inhibitory connections in the model - in terms of feed-forward vs. feed-back inhibition effects? Is it possible in their model to disentangle the contributions of FF and FB inhibition to corticospinal waves?

Feedforward and feedback effects had been disentangled by separating the analysis into afferents vs column (Fig 5F-H) but not specifically into excitation vs inhibition. To be consistent with the feedforward and feedback nomenclature, we relabeled "afferent" as "feedforward" and "column" as feedback. We additionally further separated the categories into excitatory vs inhibitory. Please see the figure below. The manuscript was updated to include this figure and the associated text (Lines 201-230).

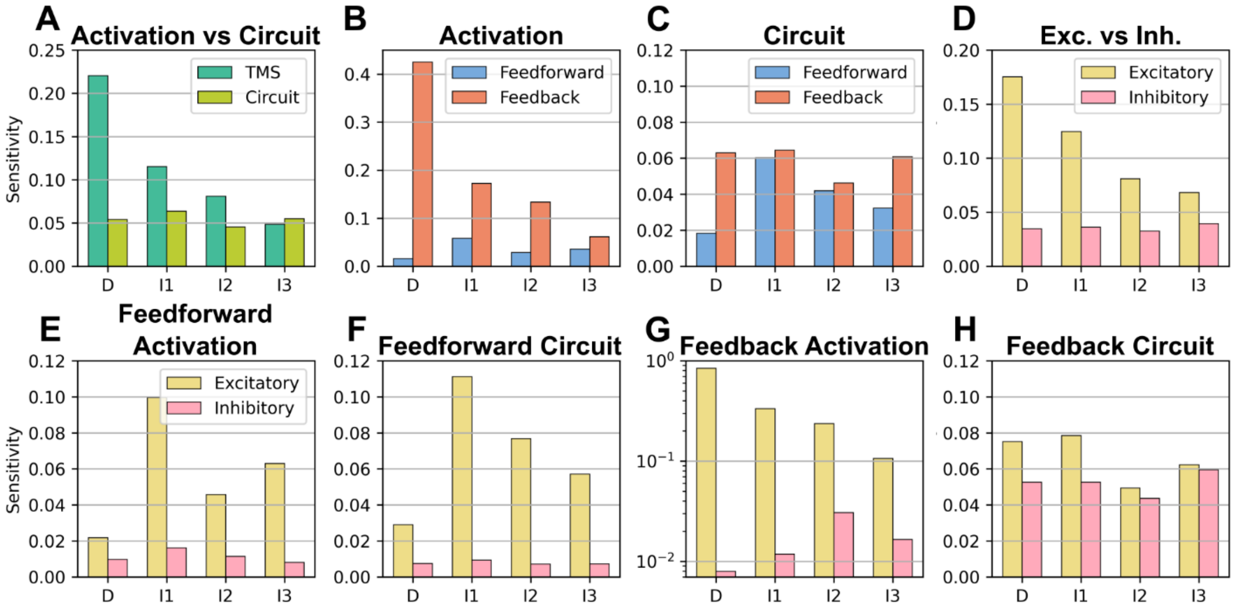

RFig. 5. *Corticospinal sensitivities. Sensitivity was computed as the average effect size for a specific corticospinal wave across all relevant mechanisms. A) Sensitivity was divided based on activation vs the synaptic strengths of the network. B) Sensitivity to activation was divided into feedforward activation, i.e., activation of extracortical afferent terminals, and feedback activation, i.e., activation of the motor cortical circuit. C) Sensitivity to the synaptic strengths was divided into the feedforward circuit, i.e., the synaptic strengths of extracortical afferents, vs the feedback circuit, i.e., the synaptic strengths of intracortical projections. D) Sensitivity was divided into elements that were excitatory vs inhibitory. E) Feedforward activation was divided into feedforward excitation, i.e., afferents targeting excitatory neurons, vs feedforward inhibition, i.e., afferents targeting inhibitory neurons. F) The synaptic strengths were divided into synaptic strengths of afferents targeting excitatory neurons vs synaptic strengths of afferents targeting inhibitory neurons. G) Feedback activation was divided into feedback excitation, i.e., activation of excitatory motor cortical neurons, vs feedback inhibition, i.e., activation of inhibitory motor cortical neurons. H) The synaptic strengths were divided into the strengths of excitatory intracortical projections vs inhibitory intracortical projections. Note the differences in y-axis values.*

"Different groupings of the total effect sizes were made to compare the average effect sizes of broader categories. The effect sizes were further subdivided based on corticospinal wave to quantify the sensitivity of the waves to the different groupings. Corticospinal waves were more sensitive to changes in activation vs changes in synaptic strength (RFig. 5A). Sensitivity was greater for activation of motor cortical neurons than activation of extracortical afferents (RFig. 5B). This was primarily driven to the large effect size of activating L5 PTNs. At the circuit level, the synaptic strengths of afferents vs motor cortical neurons had an overall similar effect (RFig. 5C). Sensitivity was greater for excitatory neurons vs inhibitory neurons (RFig. 5D). Sensitivity was greater for the activation of feedforward excitation circuits, i.e., afferents that targeted excitatory neurons (RFig. 5E). The strong sensitivity of the I1-wave is due to the effect of activating L5 PTN afferents. This is similarly reflected to the sensitivity to the synaptic strengths of afferents (RFig. 5F). Sensitivity to feedback activation, i.e., activation of motor cortical neurons, was much

stronger for excitatory neurons (RFig 5G). However, we can observe that sensitivity to inhibitory interneurons was greater in the later I-waves, I2 and I3. This is consistent with the literature. Sensitivity to synaptic strengths of intracortical projections was relatively similar across excitatory and inhibitory neurons (RFig. 5H)."

Would the addition of SOM, VIP inhibitory motifs change the dynamics of the model and possibly its conclusions (e.g. Lytton's M1 model mentioned above includes somatostatin-expressing (SOM) interneurons)? TMS-induced excitation of excitatory neurons depends on the functional state of the stimulated network, which depends on the overall inhibition mediated by the plethora of inhibitory interneurons.

The addition of SOM+ and VIP+ interneurons would affect the network dynamics. Inhibition due to these neuron types were likely compensated for during optimization by modulating the interneurons that were present, i.e., parvalbumin, fast-spiking BCs. Based on the structural analysis, the preferential contribution of SOM+ and VIP+ interneurons would be predominantly determined by their specific connectivity to L5 PTNs and the mean conduction delays in those circuits. However, there are other electrophysiological differences between SOM+ and VIP+ interneurons and BCs that could affect their contributions to the evoked response to TMS.

We acknowledge the unknown role of these interneuron types with the following text:

Lines 472-478
"Adding more neuron types is also necessary to include more types of circuits for analysis. Traditionally, L4 in motor cortex has been described as either nonexistent or very thin, which led motor cortex models to exclude L4 or represent it with inhibitory neurons only [7,30,8]. Recent evidence has identified excitatory IT neurons in L4 with projections to L2/3 [31–33] leading to more complex models of M1 [29]. The present modeling results predict that, while not included, L4 IT neurons would participate in later I-waves due to their strong projection into L2/3. Interneuron types such as SOM+, VIP+, and CCK+ would also enrich the network and allow a more complete analysis of the network response."

Minor issues:

- Line 502, 503 and line 565, 566: „The spiking activities of the afferents were generated by a Poisson process with a mean firing rate of 0.25 Hz" „the models received a random Poisson input with a mean firing rate of 1000 Hz"
A 1000 Hz Poisson input does not seem to be biologically realistic. Can you explain this and the discrepancy between the two sentences?

Because point neuron models were used, all inputs were received at the same location. For benchmarking, the inputs were condensed to a single Poisson-distributed spike train because the sum of Poisson processes produces a Poisson process with a mean that is the sum of the means of the individual Poisson processes.

A 1000 Hz Poisson train was used because it was the upper limit of the total firing rate of inputs to a neuron in the network. Although the afferents fired at 0.25 Hz, the motor cortical neurons were optimized to fire at rates between 3-15 Hz, depending on the neuron type. The average total number of inputs of each type multiplied by their respective average firing rates was approximately 1000 Hz with a range of 500-1000 Hz across all cell types.

To clarify this in the text, we added the following statement:

Lines 598-599
"This 1000 Hz firing rate was representative of the total firing rate across all inputs that a neuron would experience during a simulation used to evaluate the model response to TMS."

- The authors write: „The predictions from the model are limited to the single pulse response and are not readily extendable to paired pulse or repetitive pulse paradigms. This is partly due to GABABR parameters being underconstrained." Another important limitation is the lack of short-term synaptic plasticity mechanisms in the simple network model used. And see also https://doi.org/10.1016/j.neuron.2024.05.009 for cell-type-specific electric field entrainment, which may also affect repetitive stimulation paradigms.

The lack of STP short-term plasticity is an important point, and we included the following text.

Lines 501-504
"Finally, the model lacks a representation of short-term plasticity (STP), which contributes to non-linear facilitative and depressive effects at short-time scales. Though STP is engaged in response to paired-pulse stimuli [40], it is unknown the degree to which the series of transsynaptic activations resulting from a single pulse also contribute to I-waves, and remains an open question."
